# Identification of IKZF1 genetic mutations as new molecular subtypes in acute myeloid leukaemia

**DOI:** 10.1002/ctm2.1309

**Published:** 2023-06-21

**Authors:** Yang Wang, Wenyan Cheng, Yvyin Zhang, Yuliang Zhang, Tengfei Sun, Yongmei Zhu, Wei Yin, Jianan Zhang, Jianfeng Li, Yang Shen

**Affiliations:** ^1^ Shanghai Institute of Hematology State Key Laboratory of Medical Genomics National Research Center for Translational Medicine (shanghai) Ruijin Hospital Affiliated to Shanghai Jiao Tong University School of Medicine Shanghai China; ^2^ School of Life Sciences and Biotechnology Shanghai Jiao Tong University Shanghai China

**Keywords:** acute myeloid leukaemia, DNA binding, gene expression profiles, IKZF1, sequence variants

## Abstract

**Background:**

Genetic mutations of *IKZF1* have been frequently delineated in B‐lineage acute leukaemia (B‐ALL) but rarely elucidated in acute myeloid leukaemia (AML). *IKZF1* mutations confer a poor prognosis in AML, and hotspot mutations of *IKZF1*, N159Y and N159S tend to occur in B‐ALL and AML respectively. However, the pathogenesis of *IKZF1* N159S in AML and *IKZF1* lineage susceptibility are largely unknown.

**Methods:**

The genetic and clinical characteristics of *IKZF1*‐mutated AML patients were evaluated. Multi‐omics analysis and functional assays were performed in vitro using *IKZF1* mutations knock‐in AML cell lines.

**Results:**

23 (4.84%) small sequence variants of *IKZF1* were identified in 475 newly diagnosed AML (non‐M3) patients. Based on RNA sequencing, three classes of *IKZF1*‐related AML were defined, including 9 patients (39.13%) with *IKZF1* N159S mutations, 10 (43.47%) with *CEBPA* mutations and 4 others (17.39%). *IKZF1* N159S may define a unique subgroup with higher *HOXA/B* expression and native B‐cell immune fractions. Gene expression data of multiple knock‐in cell lines indicate that the lymphocyte differentiation‐related *MME* and *CD44* kept high expression in *IKZF1* N159Y but were downregulated in N159S. CUT&TAG sequencing showed that *IKZF1* N159S reshaped the binding profiles of IKZF1. Integration analysis suggested that the pathogenesis of *IKZF1* N159S may depend on the deregulation of several cofactors, such as oncogenic *MYC* and *CPNE7* targets.

**Conclusions:**

Collectively, we dissected the molecular spectrum and clinical features of *IKZF1‐*related AML, which may promote an in‐depth understanding of the pathogenesis, lineage susceptibility and clinical research of AML.

## Graphical abstract

Genetic mutations of *IKZF1* bared unfavourable prognostic significance in the leukaemogenesis of AML, especially for the hotspot *IKZF1* (p.N159S) point mutation.

*IKZF1* (p.N159S) point mutation occurs preferentially in AML with poor prognosis and defines a rare molecular subtype with unique gene expression.

Recurrent *IKZF1* (p.N159S) mutation exhibits a more aggressive disease progression potentially by deregulation of NOTCH signalling, VEGF, CPNE7 and MYC‐related factors.

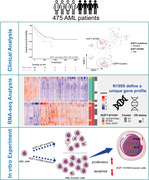

## 1 INTRODUCTION

Acute myeloid leukaemia (AML) is a group of the most aggressive haematopoietic malignancies in adults associated with development blockage and over‐proliferation of myeloid lineage cells.^1^, ^2^ Recent advances in high‐throughput sequencing and large‐scale omics studies have significantly promoted the precise molecular classification in AML, as well as in other acute leukaemia (AL). It is widely appreciated that the chromosomal abnormalities (gene fusions) and sequence variants (multi‐amino acid sites) define the major subtypes of AML,^3^, ^4^, ^5^, ^6^ B‐progenitor acute lymphoblastic leukaemia (B‐ALL)^7^, ^8^, ^9^ and T‐cell acute lymphoblastic leukaemia (T‐ALL).^10^, ^11^, ^12^, ^13^ Meanwhile, several entities with similar gene expression profiles (GEP) have been established in AL, including homologous markers, that is, fusion genes and hotspot mutations *IKZF1* p.Asn159Tyr (N159Y),^8^, ^9^ heterogeneous genetic markers, that is, *BCR::ABL1* and *BCR::ABL1*‐like pair. Recently, based on RNA‐sequencing (RNA‐Seq) data of 655 AML patients, the largest multi‐centre cohort in China, we have illuminated eight meta‐subgroups of AML, which provides a comprehensive framework to pinpoint rare molecular subtypes in AML, among which, recurrent *IKZF1* mutations was discovered in this cohort.^14^

*IKZF1* encodes IKAROS, which is a critical transcription factor for lymphopoiesis and immune haematopoiesis,^15^ and potentially works as a tumour suppressor by negatively regulating cell proliferation.^16^ IKAROS contains the N‐terminal DNA‐binding domain (DBD) with four zinc fingers (ZFs) and two additional zinc fingers situated at the C‐terminal for homo‐ and heterodimerisation.^17^, ^18^ IKAROS mainly functions as a homodimer through ZFs, but it can also dimerise with other members of the IKAROS family or various transcriptional factors to exert multiple functions,^15^, ^16^ such as nucleosome remodelling and deacetylase (NuRD) and complex polycomb repressive complex 2 (PRC2).^19^ Genetic deletions and mutations of *IKZF1* are commonly involved in the pathogenesis of ALL, especially B‐ALL. Deletion of exons 4−7 of *IKZF1* leads to the generation of Ik6 heterodimer, which interferes with normal B‐lymphocyte signalling and promotes B‐ALL development.^20^ Somatic mutations of *IKZF1* have even been more widely reported in the unfavourable molecular subtypes of B‐ALL, such as in nearly 40% of *BCR::ABL1*/‐like cases.^21^, ^22^, ^23^ In an international collaborative study of B‐ALL, we reported that *IKZF1* N159Y is a newly identified rare subtype of B‐ALL with unique gene expression profile, characterised by significant upregulation of the transcriptional coactivator *YAP1*, *SALL1* and *ARHGEF28B*, and downregulation of the B‐cell receptor signalling and JAK‐STAT signalling pathways.^24^ In addition, germline‐derived dominant negatives and haploinsufficient of *IKZF1* mutations are predisposed to T, B, and myeloid cell combined immunodeficiency and AL.^15^, ^25^

Although a large number of studies have elucidated the important pathogenic role of *IKZF1* in ALL, *IKZF1* mutations have rarely been reported in AML. A recent study reported that the frequency of *IKZF1* mutations in AML was 2.6% (5/193), which were significantly associated with *SF3B1* and bi*CEBPA* mutations.^26^ Besides, *IKZF1* mutations were retrospectively identified in OHSU (8/593, 1.35%), TCGA (1/200, 0.5%) and TARGET (4/95, 4.21%) cohorts. Among these 13 *IKZF1* mutations, 5 were N195S mutation (5/13, 38.5%).^26^ However, due to the small number of such cases and their sporadic occurrence, no targeted studies have been reported. In our previous work, we identified numerous small sequence variants of *IKZF1* with unique gene expression profiles in AML. Among them, *IKZF1* N159S was a recurrent hotspot mutation, which closely clustered in a subset of patients with myelodysplasia‐related mutations and upregulated gene expression of the *HOXA*/*B* family genes.^14^ Notably, immunomodulatory drugs inducing IKAROS protein degradation showed potential therapeutic efficiency in the *HOXA*‐related AML, which suggests the emerging synergistic role of IKAROS in high‐risk AML.^27^

To date, a few critical issues remain to be addressed to further explore the pathogenesis and improve the prognosis of AML patients with *IKZF1* mutations. Firstly, genetic alterations of *IKZF1* are common and widely reported in B‐ALL, however, the genomic landscape, molecular classification and clinical significance are scarcely investigated in a large AML cohort. Meanwhile, certain *IKZF1* mutations tend to occur in B‐ALL (p.N159Y) or AML (p.N159S) have been reported but the potential mechanism of the *IKZF1*‐related lineage susceptibility is rarely studied.^14^ Hence, in the present study, we outlined the mutational spectrum of *IKZF1* in 383 AMLs that we previously published and 92 newly diagnosed primary AMLs excluding patients with *PML::RARA* (M3). In addition, via comparison of gene expression profiles as well as in vitro investigation, we proposed to understand the underlying leukaemogenesis of *IKZF1* in AML. We hope our work will serve as a novel reference for future research on leukaemia‐lineage susceptibility, and as a starting point for a new, genetically based disease classification that will contribute to the improved risk stratification and ultimately better treatment outcomes.

## 2 METHODS

### 2.1 Ethics approval and consent to participate

Primary blasts were obtained according to the Declaration of Helsinki at disease onset from bone marrow of 475 AML patients with least 20% abnormal bone marrow blasts. RNA sequencing and targeted screening of 100 common leukaemia‐related genes was performed in all primary AML patients from Ruijin Hospital as previously described.^28^ Clinical information of 23 AML with *IKZF1* mutations is provided (Table S1). Informed consent was obtained according to procedures approved by the Institutional Review Board from Ruijin Hospital, affiliated to Shanghai Jiao Tong University School of Medicine. K562, U937, HEK‐293T and NCI‐H1299 cells were authenticated. Details are available in Supplemental Methods.

### 2.2 Other material and methods

FLAG‐tagged‐IKZF1 wild‐type or mutants were ectopically expressed in leukaemia cell line K562 and U937 using lentiviral‐mediate vector. Primers used for in vitro experimental assays are listed in Supplementary Methods. Bioinformatic/statistical analysis and other detailed information are described in Supplementary Methods.

### 2.3 Statistical analysis

Wilcoxon rank‐sum test was used for calculating the statistical significance when comparing of two groups while Kruskal‐Wallis test was used for more than two groups, paired‐samples two‐tailed Student's *t*‐test was used for estimating the statistical significance of RT‐qPCR. Data were presented as mean ± SD. All statistical analyses were performed using the R/Bioconductor statistical environment (https://www.r‐project.org/) and significance was defined as *p* < .05.

### 2.4 Role of the funding source

The funders have no role in the study design, data collection, data analysis, data interpretation or report writing.

Additional methods are outlined in Supplemental Methods.

## 3 RESULTS

### 3.1 Genomic classification of *IKZF1*‐positive AML

**FIGURE 1:**
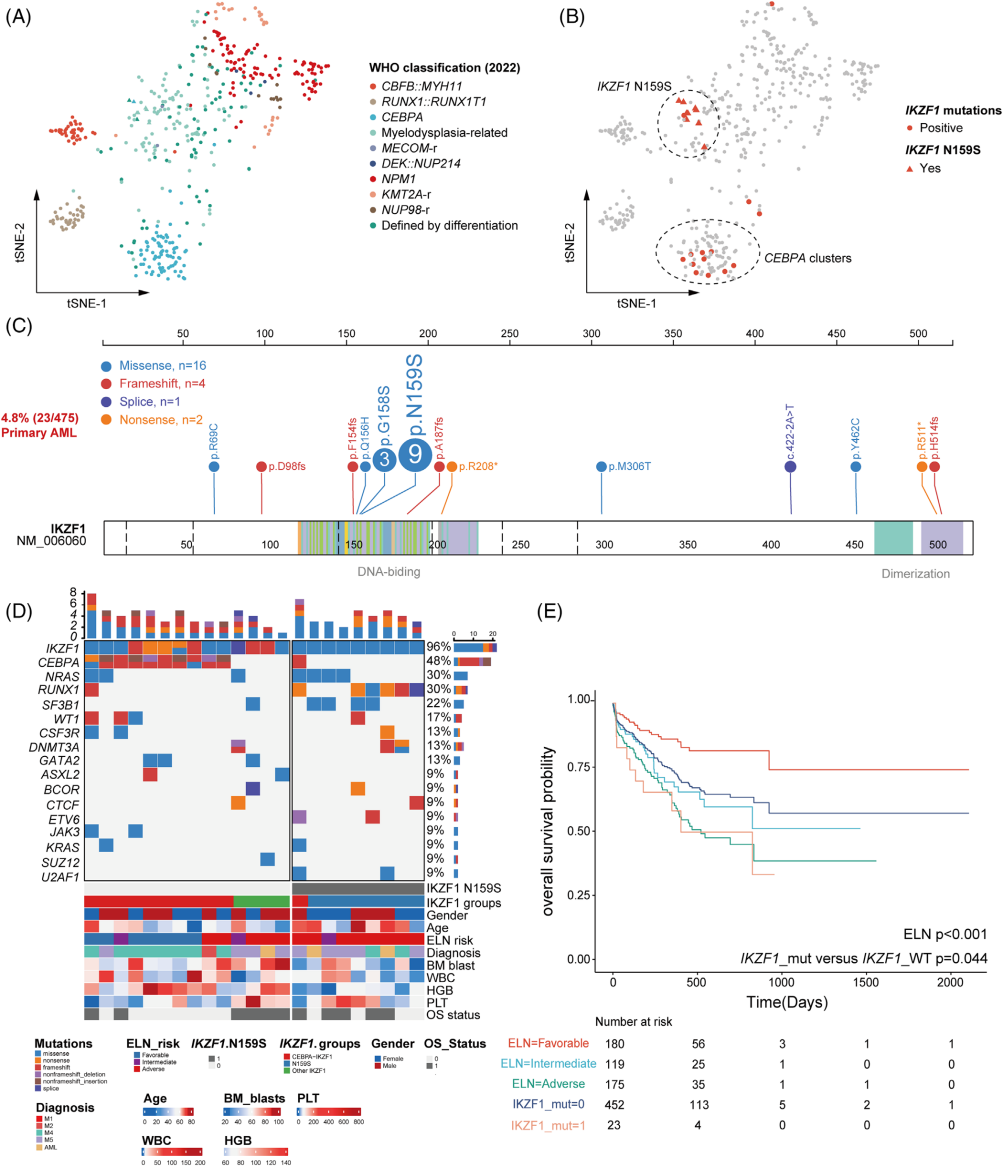
Genomic classification of *IKZF1*‐positive AML. (A) t‐SNE plots for the signatures of major somatic mutational WHO classes of our AML cohort. Each dot represents a cancer sample. Color of dots indicates cancer types or subtypes. (B) Expression clustering of representative markers for IKZF1 N159S, biCEBPA/‐like, and others AML are plotted onto the t‐SNE map. (C) Structure and mutation spectrum of *IKZF1* in AML. All *IKZF1* mutations (*n* = 23) are depicted in protein domains. Different colors represent different types of mutations. (D) Oncoplot and clinical features of patients with *IKZF1* mutations. The heatmap shows the genomic landscape and clinical features of patients with *IKZF1* mutations. (E) Overall survival of AML patients according to the ELN classes, Kaplan–Meier curves depicting the survival difference between *IKZF1*‐mutated patients and non‐*IKZF1*‐mutated patients.

The similarity of the patient samples based on gene expression data was visualised in a t‐Distributed Stochastic Neighbour Embedding (t‐SNE) analysis, which showed an overall separation of AML samples according to different genetic abnormalities defined by the 5th edition of the World Health Organization (WHO) Classification of Hematolymphoid Tumors^29^ (Figure 1A). We totally identified 23 small sequence variants of IKZF1, which comprised 4.84% (23/475) of newly diagnosed non‐M3 AML patients (Figure 1C and D; Table S1). Notably, hotspot mutations, *IKZF1* N159S occurred in 1.89% (9/475) of AML, accounting for 39.13% (9/23) of all *IKZF1* mutations in this cohort.

Consistent with our recently published work,^14^ through clustering of gene expression profiles, all cases with *IKZF1* N159S were clustered into myelodysplasia‐related/‐like (G5) subgroup, while the majority of other *IKZF1* mutant loci displayed a similar gene expression profile to biallelic *CEBPA* or ‐like (bi*CEBPA*/‐like, G4) subgroup, which incorporated not only bi*CEBPA*, but also mo*CEBPA* mutations with loss of heterozygosity and several *CEBPA* WT cases.^14^ Accordingly, three molecular subtypes with *IKZF1* aberrations were recognised including *IKZF1* N159S, bi*CEBPA*/‐like and others (Figure 1D). Furthermore, patients harbouring *IKZF1* mutations had a similar poor overall survival (OS) to those in the ELN adverse risk group (Figures 1E and S1A). Relevant clinical features suggested that haemoglobin (HGB) were significantly higher in bi*CEBPA*/‐like *IKZF1*, while bone marrow blasts, platelet (PLT) and the variant allele frequency (VAF) of *IKZF1* mutations appeared to be higher in *IKZF1* N159S‐positive cases (Figure S1B). These factors might impact the development of normal immune cells and even dim the prognosis of *IKZF1* N159S‐positive AML patients (Figure S1B and C).

### 3.2 IKZF1 N159S defines a rare molecular subtype with unique gene expression profiles in AML

**FIGURE 2:**
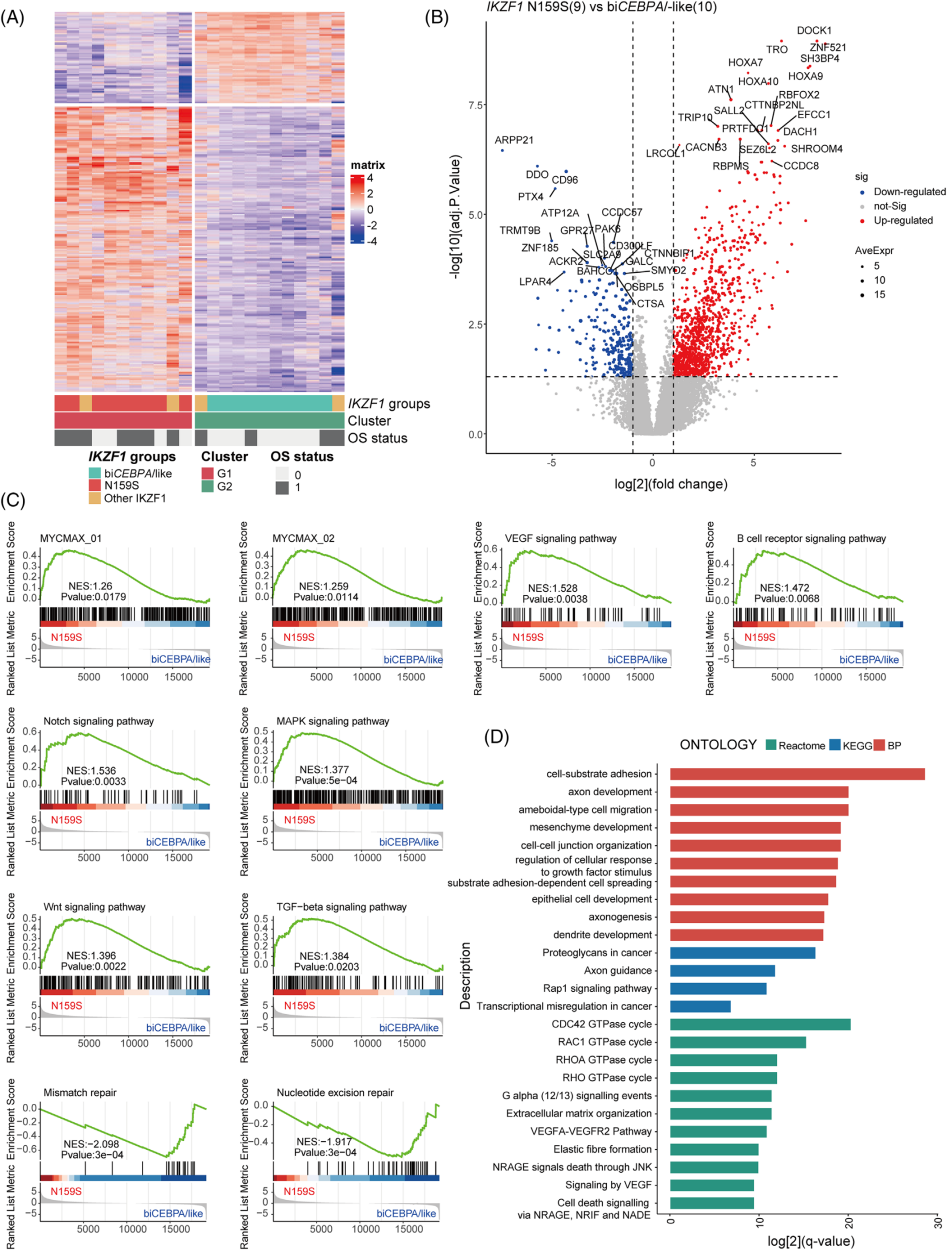
*IKZF1* N159S defines a rare molecular subtype with unique gene expression profiles in AML. (A) Unsupervised clustering using the 1267 variance genes with *p* value less than 0.05 among three classes of classifications in *IKZF1*‐positive AML. Each column represents a patient and each row represent a gene. Up‐ and downregulated genes are shown in red and blue, genomic‐based classification in *IKZF1*‐positive AML patients demonstrated that *IKZF1* N159S and bi*CEBPA*/‐like were pertained to two different subgroups, which we defined as G1 and G2, respectively. (B) Volcano plot shows the differentially expressed genes (DEGs) between *IKZF1* N159S and bi*CEBPA/‐*like cases. The *x* axis represents log2‐transformed fold‐change values, while *y* axis shows –log10‐transformed *p* value. (C) Gene set enrichment analysis of *IKZF1* N159S patients (versus bi*CEBPA/‐*like). (D) Significant positive enrichment of terms in *IKZF1* N159S‐positive AML.

Unsupervised clustering using 1267 genes with the highest variance and *p* value less than .05 confirmed the genomic‐based classification in *IKZF1*‐positive AML (Figure 2A and B). At the gene expression level, patients with *IKZF1* N159S and bi*CEBPA*/‐like *IKZF1* were mutually exclusive and pertained to two different hierarchical branches: high and low expression of *HOXA*/*B* family genes. In line with our previous work,^14^ *IKZF1* N159S was associated with myelodysplasia‐related changes, which frequently coexisted with *RUNX1* and spliceosome mutations, and was characterised by the upregulation of haematopoietic stem progenitor cell signature (HSPC) and core markers including *HOXA*/*B*, *MYCT1*, *PAWR* and *BEND4*.^14^ We hereby reproduced the above observations and extended more specific gene expression signatures of *IKZF1* N159S hotspot mutations in this work (Figures 2B and S2), that is, cytoskeletal architecture‐related (*SHROOM4*), p53‐mediated apoptosis (*CCDC8*) and activator TGF‐beta pathway (*RBPMS*). We also provided a comparison of *IKZF1*‐positive gene expression profile with other non‐*IKZF1* AML using the same 1267 genes expression, and further demonstrated that *IKZF1* N159S may define a separate molecular subtype with unique GEP in all our AML cases, even within the subcluster of myelodysplasia‐related/‐like AML (Figure S3A). Oncogenic *MYC* contributes to the genesis of many human cancers, which strictly depends on its partner Max to regulate gene transcription.^30^ GSEA analysis indicated that the MYCMAX, VEGF, B‐cell receptor, NOTCH, MAPK, WNT and TGF‐beta signalling pathways were significantly upregulated in the *IKZF1* N159S‐positive AML, while DNA mismatch repair and nucleotide excision repair pathways were significantly downregulated when compared with bi*CEBPA*/‐like *IKZF1* (Figure 2C). The Gene Ontology (GO) enrichment analysis using differentially expressed genes (DEGs) cross validated the deregulation of extracellular matrix organisation and VEGF/VEGFA signalling pathways (Figure 2D). In addition, the Rap1 signalling pathway and RHO GTPase cycle were also enriched in the *IKZF1* N159S‐positive AML based on DEGs enrichment analysis (Figure 2D). Meanwhile, the known oncogenic gene *PDGFRB* was also upregulated in the *IKZF1* N159S‐related AML, which was associated with genomic translocations demonstrating gene expression signatures of *BCR::ABL1*‐like B‐ALL.^31^ We noticed that the overall VAF distributions of *IKZF1*‐N159S tended to be slightly higher than that of *RUNX1* mutations although there was no statistical significance, suggesting that *IKZF1*‐N159S might not be secondary events to *RUNX1* mutations (Figure S3B). To evaluate the prognostic value of *IKZF1* mutations under other concomitant factors, we constructed a multivariable Cox regression model incorporating age as well as *IKZF1*, *CEBPA* and *RUNX1* mutations, and identified that mutant *IKZF1*, rather than *RUNX1*, could be an independent predictive factor for OS in AML patients (Figure S3C). Notably, the deconvolution of immune cells showed that the native B cells, T cells regulatory (Tregs), neutrophils and eosinophils were significantly higher in *IKZF1* N159S‐positive AML, which may be associated with the blocked differentiation of B lymphocytes and specific regulation of microenvironment by *IKZF1*‐positive tumour cells (Figure S4).

### 3.3 Functional effects of *IKZF1* variants G158S, N159Y and N159S in vitro

**FIGURE 3:**
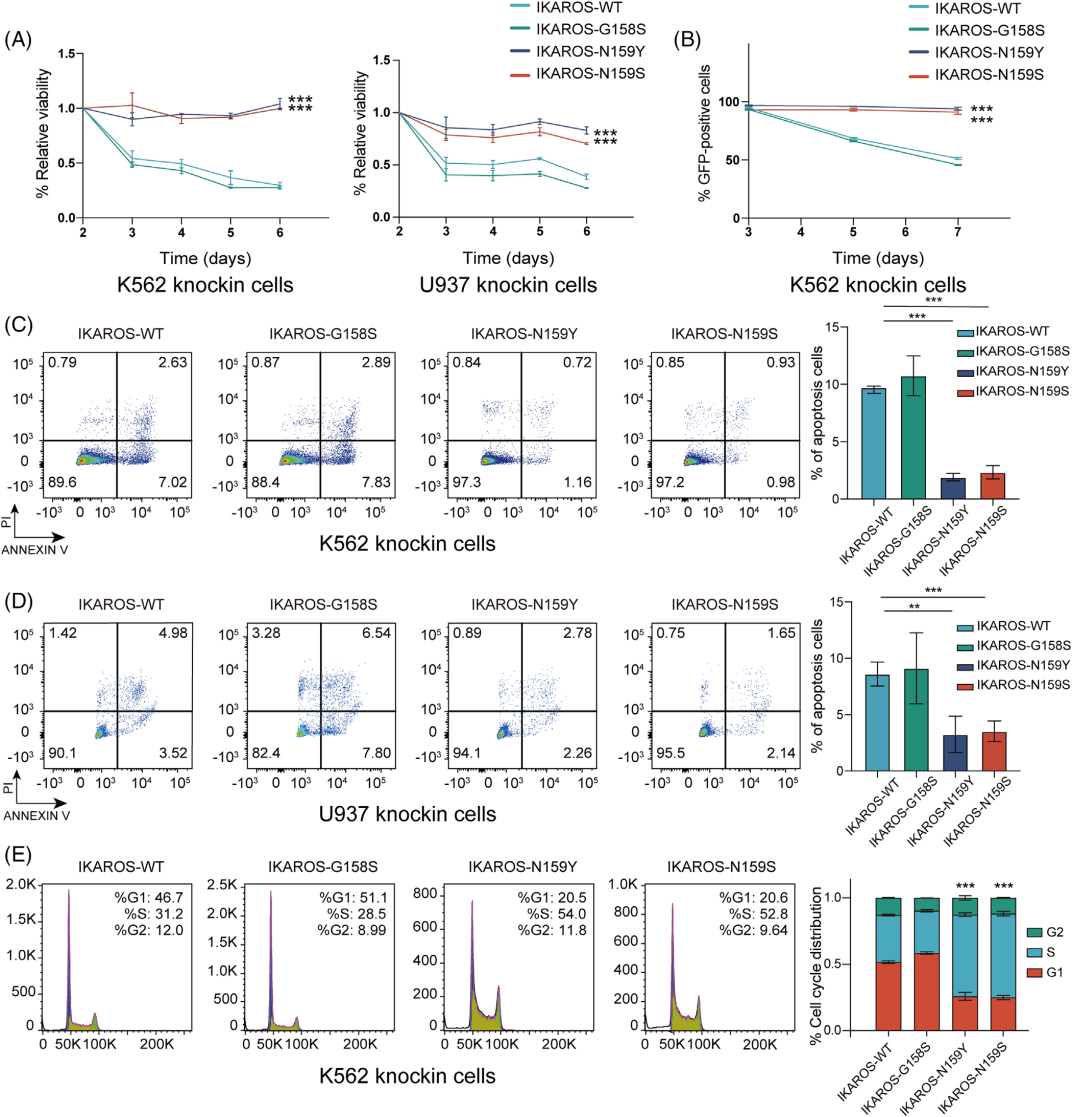
Functional effects of *IKZF1* variants. (A) CCK8 assays demonstrating the proliferative capacity of K562 and U937 following transfection with various *IKZF1* mutation, normalised to native K562 and U937. (B) Negative‐selection competition assays showing the percentage of various GFP+ *IKZF1* mutants‐transduced K562 over time, as normalised to Day 3. (C) Apoptosis analysis in *IKZF1* mutants‐expressing K562. *IKZF1* WT and *IKZF1* G158S induced apoptosis, as measured by annexin V/PI flow cytometric analyses, while *IKZF1* N159S/Y defect the ability to induce apoptosis. The histogram depicts the quantification of total proportion of cells in early (annexin V+/PI–) and late apoptotic (annexin V+/PI+) stages. (D) Apoptosis analysis in *IKZF1* mutants‐expressing U937 cells. (E) Cell cycle analysis of *IKZF1* mutants‐expressing K562 cells. *IKZF1* WT and *IKZF1* G158S induce cell cycle arrested at G0/G1 phase, while *IKZF1* N159S/Y defect the ability to arrest the cell cycle. The histogram depicts DNA content of cell cycle progression in *IKZF1* mutants‐expressing K562 cells. All plots are representative of at least three independent experiments performed in duplicate and presented as the means ± standard deviation. (*p < .05, **p < .01 and ***p < .001).

To evaluate the function of highly recurrent *IKZF1* variants in a cellular context, we performed in vitro experiments to assess the impact of different variants on IKAROS activities and leukaemia cell phenotypes. Two most frequent point mutations in our cohort including *IKZF1* N159S and G158S were chosen for further investigation, and *IKZF1* N159Y was also selected as a positive control, which was extensively reported to affect the initiation and development of ALL and disturb the normal function of wild‐type (WT) IKAROS.^32^, ^33^ Ectopic expression of WT *IKZF1* resulted in significant inhibition of cell growth and proliferation coinciding with its tumour‐suppression function, as measured by CCK‐8 assays in leukaemia cells K562 and U937. In contrast, cells with N159S and N159Y expression showed more aggressive cell proliferation; however, G158S expression cells showed a similar growth level to that of the WT *IKZF1* cells, suggesting that G158S potentially retains the normal tumour‐suppression function of IKAROS, while N159S/Y could partially impair this function (Figure 3A). Results of GFP relative depletion further supported the CCK‐8 assays (Figure 3B). Flow cytometry of annexin V/PI staining was performed to evaluate the effects of various *IKZF1* mutants on apoptosis of K562 and U937 leukaemia cells, which suggested that WT IKAROS and G158S could induce more apoptosis as compared with N159S and N159Y (Figure 3C and D). Cell cycle analysis by flow cytometry showed that in comparison with N159S/Y‐positive cells, more WT and G158S cells arrested in the G1 phase, resulting in fewer cells in the G2/M stage (Figure 3E). These results suggested that *IKZF1* N159S might disturb the normal function of IKAROS and relieved the tumour‐suppression effects of wild‐type IKAROS.

### 3.4 Comparative analysis of *IKZF1* mutant cell lines

**FIGURE 4:**
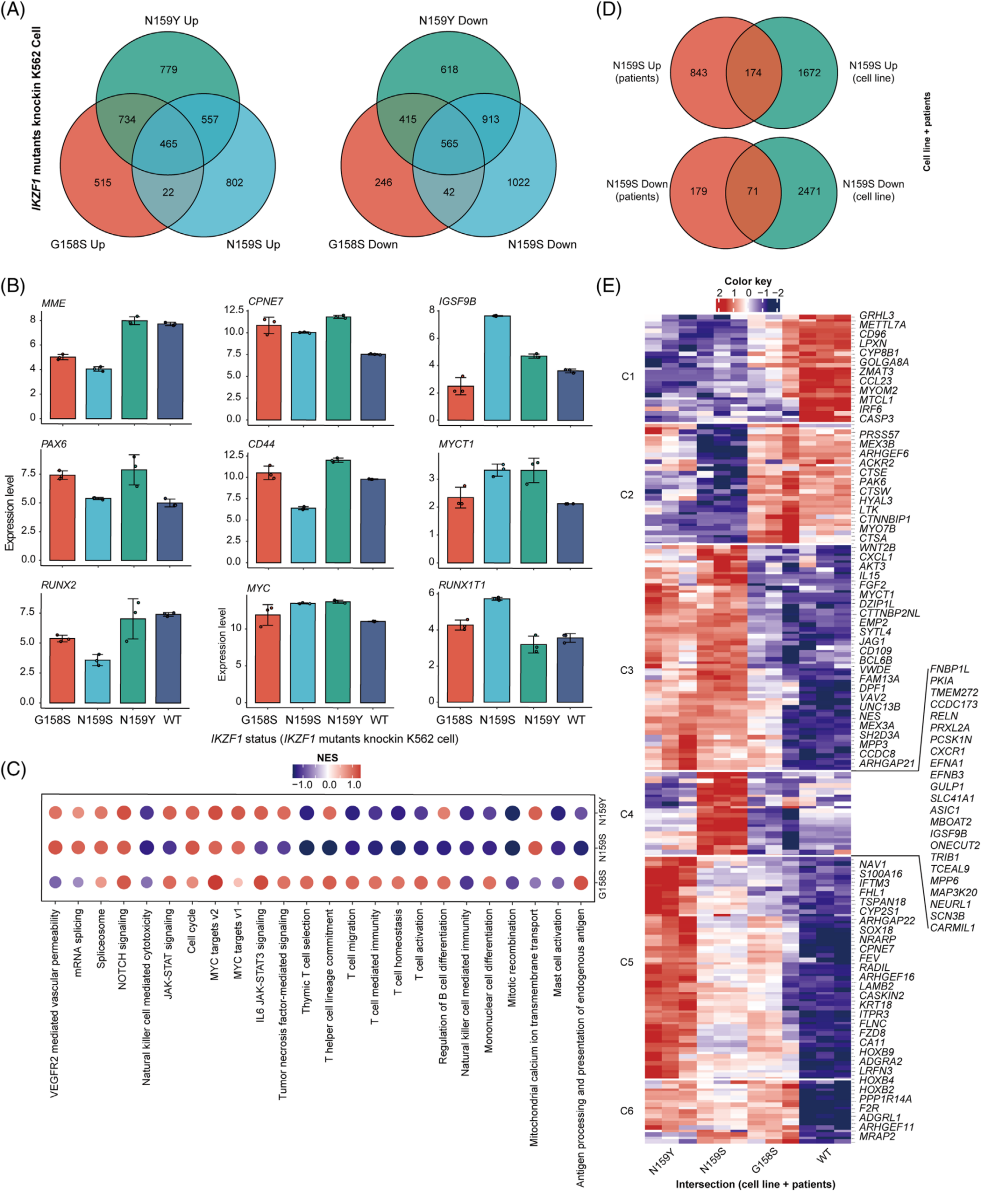
Bulk RNA‐Seq analysis of *IKZF1* WT, G158S, N159S and N159Y knock‐in human cell lines define codrivers of *IKZF1* N159S‐positive AML. (A) Venn diagram depicting the relationship of differentially up‐ and downregulated expressed genes (DEGs) between *IKZF1* G158S, N159S and N159Y mutation knock‐in K562 cell line (versus WT). (B) Bar plots showing the expression level of 9 selected DEGs in the RNA‐Seq data. (C) Dot plot detailing the enriched pathways of *IKZF1* G158S, N159S and N159Y mutation knock‐in K562 cell line. (D) Venn diagram depicting the relationship of differentially expressed genes (DEGs) between *IKZF1* N159S‐positive AML and *IKZF1* N159S knock‐in K562 cell line. (E) Heatmap showing the hierarchical clustering of *IKZF1* G158S, N159S and N159Y mutation knock‐in K562 cell line (versus WT) based on DEGs intersection of *IKZF1* N159S‐positive AML and *IKZF1* N159S knock‐in cell. Gene signatures of *IKZF1* N159S can be divided into six subclasses, namely, C1–C6. These subclusters of genes are enriched in WT, WT/G158S, N159Y/S, N159S and N159Y, respectively.

According to the published genomic data from thousands of patients with acute leukaemia, we noticed that *IKZF1* N159Y and N159S were almost mutually exclusive in B‐ALL and T‐ALL/AML.^24^, ^26^ To screen the potential lineage specific gene expression patterns following *IKZF1* mutations, we then performed bulk RNA‐Seq of *IKZF1* G158S, N159S and N159Y mutant human cell lines (Table S2). Unsupervised clustering of mutant and WT *IKZF1* cell lines exhibited that different changes at the same amino acid position of *IKZF1* or at different positions can produce distinct gene expression profiles (Figures 4A and S5). *MME*, a well‐known cell surface marker of B‐ALL, showed high expression in *IKZF1* N159Y, which was close to WT phenotype, but downregulated in *IKZF1* N159S/G158S (Figure 4B). Several specific downregulated genes in *IKZF1* N159Y/S may also contribute to the lineage susceptibility, as exemplified by *CD44*, *PAX6*, *RUNX2*, *IGSF9B* and *RUNX1T1* (Figure 4B). GSEA analysis indicated that NOTCH signalling, cell cycle, and MYC targets pathways were commonly upregulated in *IKZF1* mutants, suggesting the tumour suppressor function of *IKZF1*. On the contrary, mitotic recombination and development of natural killer cell/mast cell were consistently downregulated in all three *IKZF1* mutants (Figure 4C). Meanwhile, T‐cell immune regulation, antigen processing/presentation and mononuclear cell differentiation were downregulated in both *IKZF1* N159S and N159Y. B‐cell differentiation and JAK‐STAT signalling were only downregulated in *IKZF1* N159S mutant cell line (Figure 4C). When focusing on the DEGs of *IKZF1* N159S (versus WT), the deregulated gene sets can be divided into six subclasses, namely, C1‐C6. These subclusters of genes were enriched in WT, WT/G158S, N159Y/S, N159S and N159Y, respectively. Among them, we identified at least 23 genes, that is, *RELN*, *CXCR1*, *IGSF9B*, *ONECUT2*, *TRIB1* and *MAP3K20*, that were specifically upregulated in N159S mutant cell line (Figure 4E).

### 3.5 *IKZF1* N159S reshapes its genome binding patterns

**FIGURE 5:**
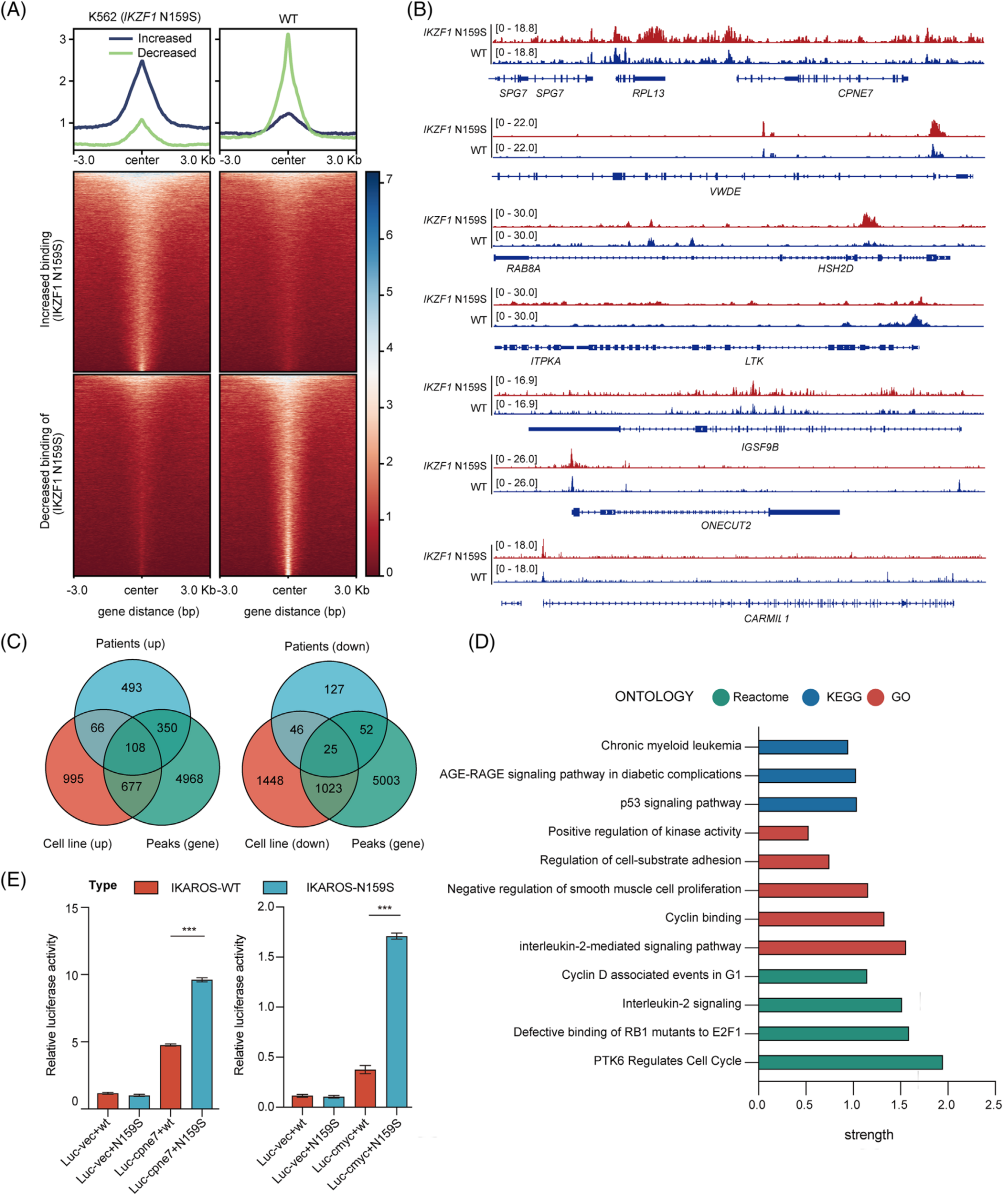
IKZF1 N159S reshapes its genome binding patterns. (A) Distribution of reads in differentially binding peaks in *IKZF1* WT and *IKZF1* N159S knock‐in K562 cell line. The upper panel shows a line plot depicting the intensity of IKZF1 WT and IKZF1 N159S binding signals centred at the peaks. The lower heatmap shows signals at and ± 3 kb the centre of the peaks. (B) Tracks showing the IKZF1 WT and IKZF1 N159S occupancy at genomic loci promoter and/or enhancer regions of selected target genes based on combination analysis of RNA‐Seq and CUT&TAG sequencing data. (C) Venn diagram depicting the intersection between differentially expressed genes (DEGs) of *IKZF1* N159S‐positive patients, *IKZF1* N159S knock‐in K562 cell line and CUT&TAG binding peaks. (D) Significant positive enrichment of terms in intersection gene sets based on Protein‐protein interaction (PPI) network analysis. (E) Luciferase activity assay detected the transcriptional regulation region of *MYC* and *CPNE7* bound by IKZF1 WT and IKZF1 N159S. 1299 cells were transfected with the *MYC* and *CPNE7* luciferase reporter plasmid and predicted *IKZF1* WT and *IKZF1* N159S mimics. The luciferase activity was detected 48 h after transfection in 1299 cells. The plots are representative of at least three independent experiments performed in duplicate and presented as the means ± standard deviation (*p < .05, **p < .01 and ***p < .001).

To explore the genomic binding profiles of the most significant hotspot mutation *IKZF1* N159S in AML and recognise direct gene targets of IKZF1 N159S, CUT&TAG sequencing was performed in the *IKZF1* N159S mutant cell line (Table S2). Compared with *IKZF1* WT, the genome binding pattern was reshaped by *IKZF1* N159S, which was in line with the variations of gene expression profiles (Figure 5A). These differential binding peaks may drive the common GEP of *IKZF1* mutants, that is, *CPNE7*, *VWDE*; and specific signatures of *IKZF1* N159Y/S, that is, *HSH2D*, *LTK*; and *IKZF1* N159S, that is, *IGSF9B*, *ONECUT2* and *CARMIL1* (Figure 5B). When compared with DEGs of *IKZF1* N159S‐positive AML, we screened 108 upregulation and 25 downregulation of *IKZF1*‐related DEGs, which showed consistent trend in RNA‐Seq data of patients and cell lines (Figure 5C). A protein interaction network was constructed to integrate and visualise these screened downstream factors of *IKZF1* N159S (Figure S6), functional enrichment analysis indicated that the interacting proteins were significantly enriched in pathways related to cancer development, that is, P53 signalling pathways, and were also enriched in the cell cycle regulation‐related pathways, consistent with our previous function assays (Figure 5D). We finally focused on the genes that are differentially regulated by *IKZF1* N159S both in patient cells, cell line RNA‐Seq and CUT&TAG data. *CPNE7*, a member of calcium‐dependent proteins family that was rarely reported in acute leukaemia, was commonly regulated by *IKZF1* mutations and was significantly occupied by *IKZF1* N159S rather than WT *IKZF1*. Hence, *CPNE7* might be one of the common underlying factors possibly responsible for the high pathogenicity of *IKZF1* mutations. Moreover, another member of the same gene family *CPNE8* has been defined as the coexpression factor of *HOXA*/*B*, which could predict a significantly poor prognosis in AML.^14^ To assess whether *CPNE7* could be directly activated by *IKZF1* N159S rather than WT *IKZF1*, we performed the luciferase experiments. The luciferase activity was higher in cells transfected with the *IKZF1* N159S mimics and *CPNE7* than in those transfected with the WT *IKZF1* mimics and *CPNE7* (Figure 5E, left panel). We also conducted the luciferase experiments focusing on *MYC*, the specific gene signature of *IKZF1* N159Y/S (Figure 4B), which has been reported to be a target gene of *IKZF1* and directly suppressed by WT *IKZF1*.^25^, ^34^, ^35^ *MYC* was significantly activated by *IKZF1* N159S rather than *IKZF1* WT (Figure 5E, right panel), which further confirmed the leukaemogenic effect of *IKZF1* N159S mutation.

### 3.6 *IKZF1* N159S strengthen the expression of *MYC* and *CPNE7* to promote carcinogenesis

**FIGURE 6:**
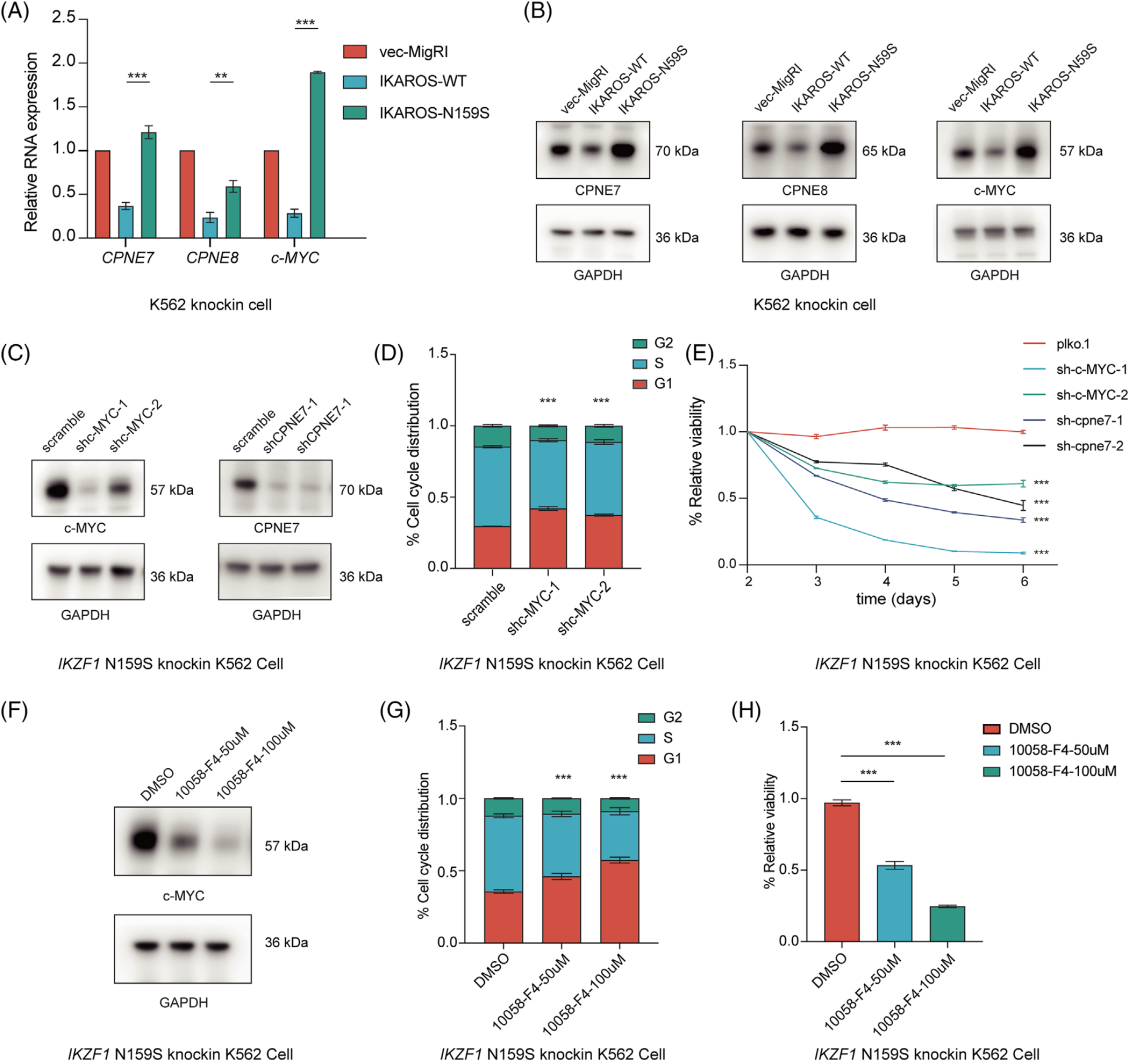
*IKZF1* N159S mutation strengthen the expression of *MYC* and *CPNE7* to promote carcinogenesis. (A) Qpcr validation of differential gene expressions. The graph depicting relative expression level of *MYC*, *CPNE7* and *CPNE8* mRNA in *IKZF1* WT and *IKZF1* N159S expressing K562 cells 5 days post lentiviral transduction, and gene expression level was normalised to the reference gene *GAPDH*. (B) Western blot of MYC, CPNE7 and CPNE8 in empty vector, *IKZF1* WT and *IKZF1* N159S transduced K562 cells 5 days post lentiviral transduction. (C) Western blot detection of MYC and CPNE7 in *IKZF1* N159S expressing K562 cells after transducing with lentivirus expressing either a scramble (control) shRNA or two independent shRNAs targeting at *MYC* and *CPNE7*. Cells were selected and immunoblotted at 4 days post‐transduction. (D) Cell cycle distribution calculation of *IKZF1* N159S expressing cells after knocking down *MYC*, a growing proportion of cells arrested in G1 phase after *MYC* knockdown. Cell cycle distribution assayed as described in the Methods section. (E) Cck8 proliferation assay of *IKZF1* N159S expressing cells control versus *MYC* or *CPNE7* knockdown, *MYC* or *CPNE7* knockdown inhibit the proliferation ability of *IKZF1* N159S expressing cells. The viability of cells was measured daily beginning 3 days post‐transduction of shRNAs. (F) Western blot analysis of MYC protein level in *IKZF1* N159S expressing cells treated with 10058‐F4 for 24 h at the concentrations labelled, *GAPDH* served as a loading control. MYC protein expression is negatively regulated by 10058‐F4. (G) Flow‐cytometry‐based quantification of cell cycle distribution of *IKZF1* N159S expressing cells treated with 10058‐F4, with increasing drug concentration, more and more cells are blocked in G1 phase. (H) Cck8 assay demonstrated the dose‐dependent effects of 10058‐F4 in the proliferation capability of *IKZF1* N159S expressing cells, cell proliferation was inhibited with increasing drug concentration. All plots are representative of at least three independent experiments performed in duplicate and presented as the means ± standard deviation (*p < .05, **p < .01 and ***p < .001).

According to the aforementioned analysis, consistently, a significant upregulation of *MYC, CPNE7* and *CPNE8* mRNA was observed in *IKZF1* N159S‐expressing cells when compared to *IKZF1* WT‐expressing cells (Figure 6A). In parallel, *IKZF1* N159S was accompanied by increased expression levels of *MYC*, *CPNE7* and *CPNE8* proteins in contrast to that in *IKZF1* WT (Figure 6B). *MYC*, a multifunctional transcription factor protein, behaved as a common gene signature upregulated by *IKZF1* N159Y/S,^25^, ^34^ and contributed to the pathogenesis of various types of human cancers through different mechanisms.^36^, ^37^ Upregulation of *MYC* following *IKZF1* N159S may herald underlying pathogenesis in this molecular subtype. In addition, the *CPNE* gene family are calcium‐dependent phospholipid‐binding proteins with intrinsic kinase activity, although the exact biological functions of *CPNE* family proteins remain unclear, an increasing number of studies have shown that *CPNE* family proteins may mediate various signalling pathways involved in tumourigenesis and progression.^38^ Therefore, further investigation on the synergistic pathway of *IKZF1* N159S‐*CPNE7* may provide new insights into the pathogenesis and refine the treatment of AML. Based on the theoretical assumptions above, we designated the rescue experiment aiming at *IKZF1* N159S‐expressing cells, and short hairpin RNAs (shRNAs) targeting *MYC* and *CPNE7* were used to knockdown the expression level of *MYC* and *CPNE7* in N159S‐expressing cells (Figure 6C). Decreased expression of *MYC* in *IKZF1* N159S‐expressing cells was typically associated with a subsequent G1 cell cycle arrest (Figure 6D). Meanwhile, both *MYC* and *CPNE7* depletion in *IKZF1* N159S cells blocked cell growth detected by CCK‐8 assays (Figure 6E). To further exploit potential therapeutic options for patients with *IKZF1* N159S mutation, 10058‐F4, an appealing inhibitor of *MYC* was applied in our research, which was reported to exert remarkable anti‐cancer capability in hepatocellular carcinoma,^39^ prostate cancer^40^ as well as various haematologic malignancies.^41^, ^42^ Western blot analysis of 10058‐F4‐treated *IKZF1* N159S cells revealed dose‐dependent inhibition of *MYC* protein after 24 h (Figure 6F). A concentration‐dependent increase in the fraction of cells in the G1 phase was detected after 24‐h period of drug exposure (Figure 6G). CCK‐8 assays showed dose‐dependent inhibition of viability of *IKZF1* N159S cells that in parallel with G1 cell cycle arrest (Figure 6H). These results suggested that *IKZF1* missense mutation N159S may augment the biological function of a series of oncogenes, among which, the engagement of *MYC* and *CPNE7* might play a pivotal role in *IKZF1* N159S‐mediated tumourigenesis. Considering *MYC* is a proliferation‐related gene, a direct effect of inhibitor 10058‐F4 or MYC shRNA should not be neglected. Genetic ablation or pharmaceutical blockade of *MYC* or *CPNE7* could potentially provide innovative treatment options for AML patients with *IKZF1* N159S mutation.

## 4 DISCUSSION

Acute myeloid leukaemia (AML) is a group of highly genetic heterogeneous diseases with various prognosis. Functionally, gene mutations could be classified into different types, as exemplified by the dysregulation of transcription factors (TFs), that is, *CEBPA, RUNX1* and *IKZF1* and epigenetic regulators, that is, *DNMT3A*, *IDH1/2* and *ASXL1*, which are indispensable for the control of myeloid progenitor cell differentiation and lineage determination.^34^ *IKZF1*, a well‐known tumour suppressor and transcription factor in haematopoiesis, plays a critical role in lymphoid differentiation, as well as in myeloid development. Several mouse models with constitutive or conditional *IKZF1* knockout exhibited disturbance of normal lymphoid and myeloid differentiation, and developed aggressive diseases such as widespread haematopoiesis failure, thymic lymphoma or T‐cell malignancies.

With the advent of the era of high‐throughput sequencing and precision medicine of AL, genetic mutations of *IKZF1* were firstly reported in aggressive B‐ALL subtypes, that is, *BCR::ABL1*/‐like, which were characterised by the high relapse rate and short survival duration.^8^, ^16^, ^25^ However, *IKZF1* mutations were poorly characterised in the research field of AML. Only a few studies have reported the rare *IKZF1* mutations in acute myeloid leukaemia with a low frequency.^26^, ^43^ On the other hand, the lineage plasticity refers to the ability of transition from one committed developmental pathway to another,^44^ relapse can be associated with a lineage switch from acute lymphoblastic to acute myeloid leukaemia, which may be resistant to chemo‐ and immunotherapies and result in poor clinical outcomes.^45^ Previous studies have shown that the lineage switching is linked to a major rewiring of gene regulatory networks in patients with *KMT2A* translocations.^34^, ^44^, ^45^ But the potential role of *IKZF1* (IKAROS) in disease fate determination has been rarely explored.

Herein, we conducted a genomics/transcriptomics analysis in 475 newly diagnosed cases of non‐M3 AML. The genomic landscape of *IKZF1* sequence variants (*n* = 23) in AML was elucidated. Three major classes of *IKZF1* mutations were identified including *IKZF1* N159S (*n* = 9, 39.13%), bi*CEBPA*/‐like *IKZF1* (*n* = 10, 43.47%) and others (*n* = 4). Most of them may result in abnormal genome binding patterns and gene expression regulations via impacting the DNA‐binding domain of *IKZF1*. Besides, the hotspot *IKZF1* N159S mutations frequently coexisted with *RUNX1* and spliceosome mutations. These comutations are common in the myelodysplasia‐related/‐like AML, particularly in elderly populations. Prognosis‐wise, AML patients with *IKZF1* N159S had an extremely poor prognosis, even worse than *TP53* mutation/complex karyotype AML in our cohort. According to the available drug response data, combination therapy of demethylation agents and Homoharringtonine (HHT)/all‐trans‐retinoic acid (ATRA) showed potential sensitivity in *IKZF1* N159S‐positive AML (Table S1). Above all, incorporating *IKZF1* into the prognostic stratification of AML may improve the diagnosis discrimination and facilitate the development of new tailored therapies for AML. Meanwhile, we also noticed that the *IKZF1* mutations coexisted with bi*CEBPA* are associated with more favourable prognosis, like *DUX4* fusions in B‐ALL, which could reverse the negative effect of *IKZF1* abnormalities. The related factors still warrant further exploration by more comparative functional studies.

Gene expression profiles indicated that compared with bi*CEBPA*/‐like *IKZF1*, the *HOXA*/*B*, HSPC expression signature, proto‐oncogene pathways including VEGF, B‐cell receptor, NOTCH, MAPK, WNT, TGF‐beta, Rap1 signalling pathways were significantly upregulated in the *IKZF1* N159S‐positive AML. Activated NOTCH signalling was also observed in T‐ALL with *IKZF1* N159S.^14^ Moreover, the known kinase signalling factor (*PDGFRB*) of *BCR::ABL1*‐like B‐ALL was also upregulated in *IKZF1* N159S‐related AML.^31^ In contrast, the DNA repair‐related pathways were significantly downregulated in patients with *IKZF1* N159S. These specifically enriched pathways may partially interpret the unique immune microenvironment and clinical prognosis of *IKZF1* N159S, providing a wealth of therapeutic targets/pathways for this high‐risk AML subtype.

Next, we performed in vitro functional experiments to investigate the cellular effects of *IKZF1* genetic mutations. *IKZF1* N159S exerted aberrant regulation of cell apoptosis, cell cycle and cell viability, which indicated the perturbation of the tumour‐suppression function of *IKZF1*. We then compared the gene expression profiles of hotspot mutations *IKZF1* N159S, G158S and N159Y and found that B‐lymphoid development markers, such as *MME* and *CD44*, were highly expressed in N159Y‐positive but downregulated in N159S‐positive cell line. *RUNX2* was also highly expressed in *IKZF1* N159Y knock‐in AML cells, which was reported to be abundant in the haematopoietic stem/progenitor cells in mice, and the upregulation of *RUNX2* led to the repression of myeloid differentiation.^46^ Conversely, *RUNX1T1*, a well‐known factor for haematopoietic differentiation and myeloid development, was highly expressed in *IKZF1* N159S knock‐in AML cells instead of *IKZF1* N159Y.^47^ These data suggested that the dysregulation of gene transcriptional profiles may underpin the fundamental lineage reprogramming.^45^ We therefore propose that different regulation effect of genetic mutations at the same amino acid site of transcription factors may lead to different regulation effect and leukaemia lineage.

Based on gene expression profiling and CUT&TAG sequencing of knock‐in human cell lines, we identified several potential common and specific pathways, that is, JAK‐STAT, NOTCH signalling pathway and B‐cell development, and genes, that is, *CPNE7*, *VWDE*, *LTK* and *IGSF9B*, of *IKZF1* N159S‐positive AML. Among these, we speculate that the inherent characteristics of recurrent *IKZF1* N159S mutation might lead to a more deteriorative evolution of AML via upregulating *MYC* targets and *CPNE7*, which were directly regulated by IKZF1 N159S. Inhibitors of *MYC* have been widely exploited, that is, 10058‐F4, APTO‐253. Therefore, the employment of MYC inhibitors could bring new therapeutic ideas for patients with *IKZF1* N159S mutations.

Taken together, our data identified the mutation spectrum of *IKZF1* and defined the significant function of *IKZF1* N159S in AML. The clinical and biological significance of *IKZF1* variants were explored both in patients and cell line level, highlighting the key role of *IKZF1* in the pathogenesis, treatment, and stratification of AML. Furthermore, it may appear as an important molecular target for tailored treatment, as exemplified by *MYC* inhibitor. These findings may further promote the accurate classification and improvement of prognosis for patients with *IKZF1*‐positive AML, which may also exert potential impact on other tumour researches involving immune microenvironment and lineage switching mechanism.

## AUTHOR CONTRIBUTIONS

SY and LJF conceived and designed the study. WY and ZYY performed the experiments. WY, LJF and ZYL analysed and interpreted data. CWY, WY, STF, YW, ZJN and ZYM collected the clinical data; WY and LJF wrote the manuscript. CWY, ZYY and SY revised the paper. All authors reviewed the data and paper and approved the final version of the manuscript.

## FUNDING

This work was supported by the National Natural Science Foundation of China (No. 82270116) and the Shanghai Municipal Education Commission‐Gaofeng Clinical Medicine Grant Support (No. 20161406).

## CONFLICT OF INTEREST STATEMENT

The authors declare no competing interests.

## Supporting information

Supporting Information

Supporting Information

Supporting Information

Supporting Information

Supporting Information

Supporting Information

Supporting Information

Supporting Information

Supporting Information

## Data Availability

Cell line sequencing data have been deposited into the GSA‐Human database (https://ngdc.cncb.ac.cn/gsa‐human/) under accession identification numbers subHRA004087. All data sets generated and/or analysed during the current study are available from the corresponding author on reasonable request. Please contact yang_shen@sjtu.edu.cn.
