# Supplementary figures and images for: Identification of IKZF1 genetic mutations as new molecular subtypes in acute myeloid leukaemia
Source: Clin Transl Med. 2023 Jun 21;13(6):e1309. doi: 10.1002/ctm2.1309 (PMC10285267; doi:10.1002/ctm2.1309)

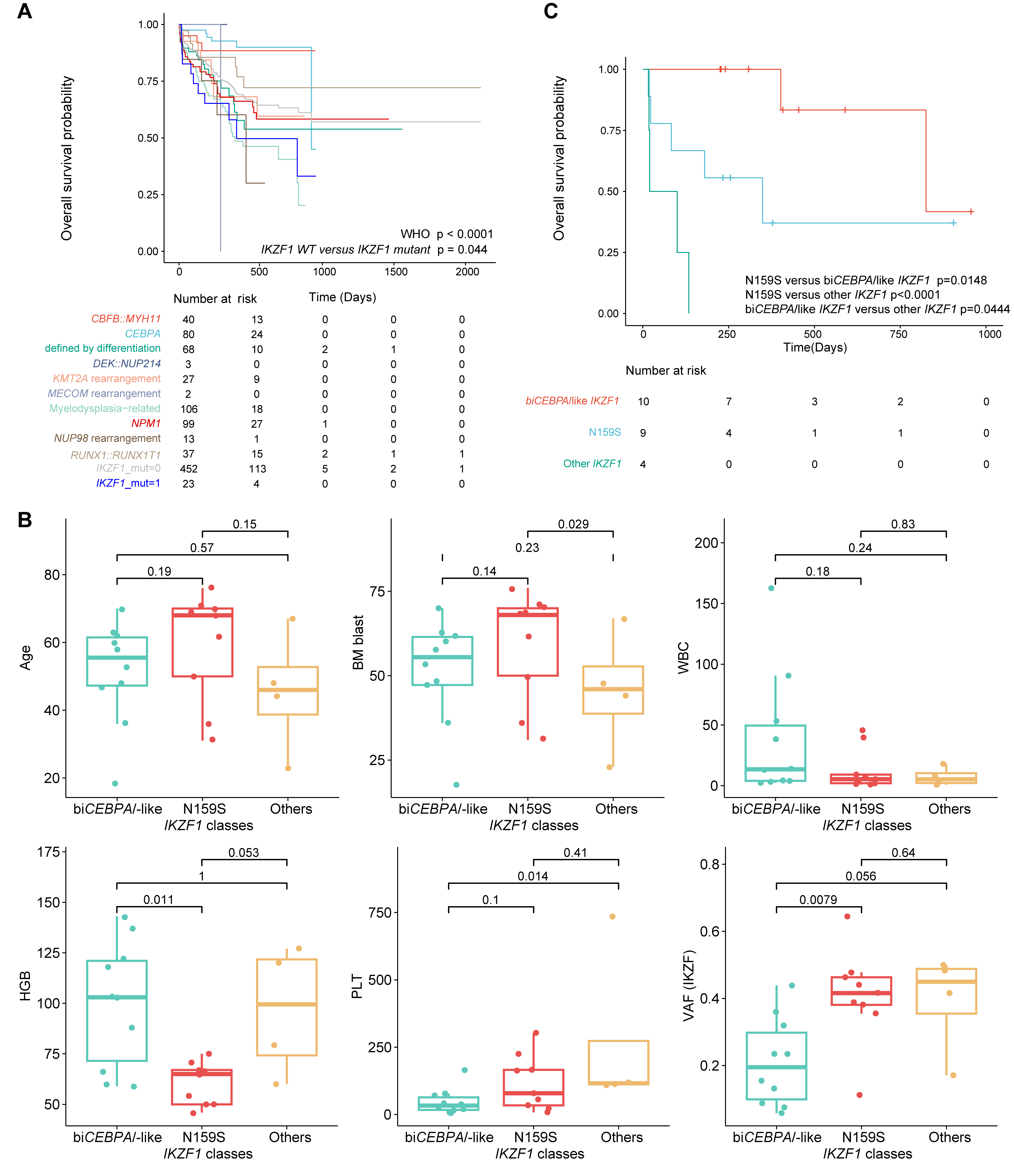

Supplement: Supplementary file 3 — Supporting Information [file CTM2-13-e1309-s007.tif]

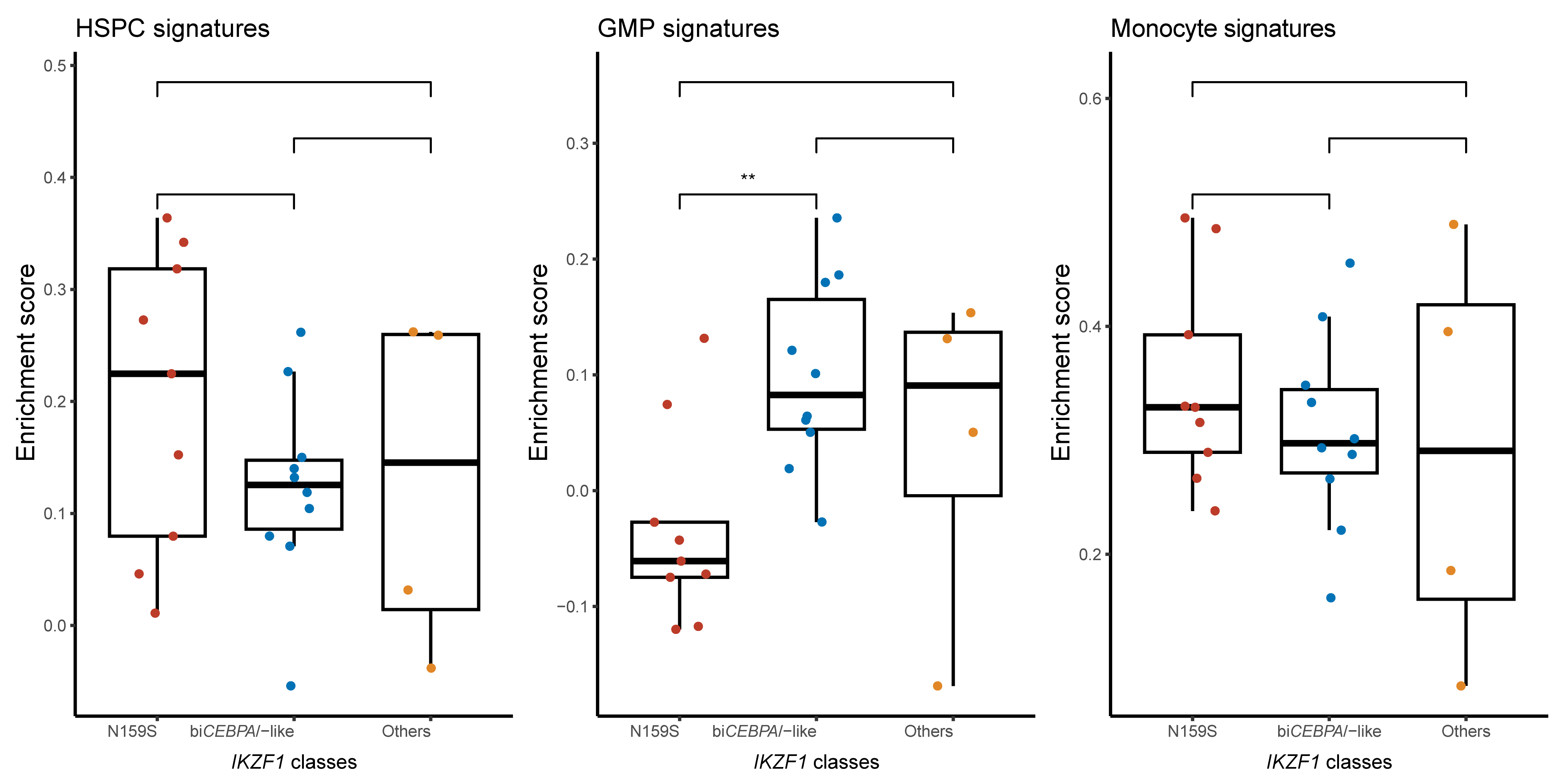

Supplement: Supplementary file 4 — Supporting Information [file CTM2-13-e1309-s008.tif]

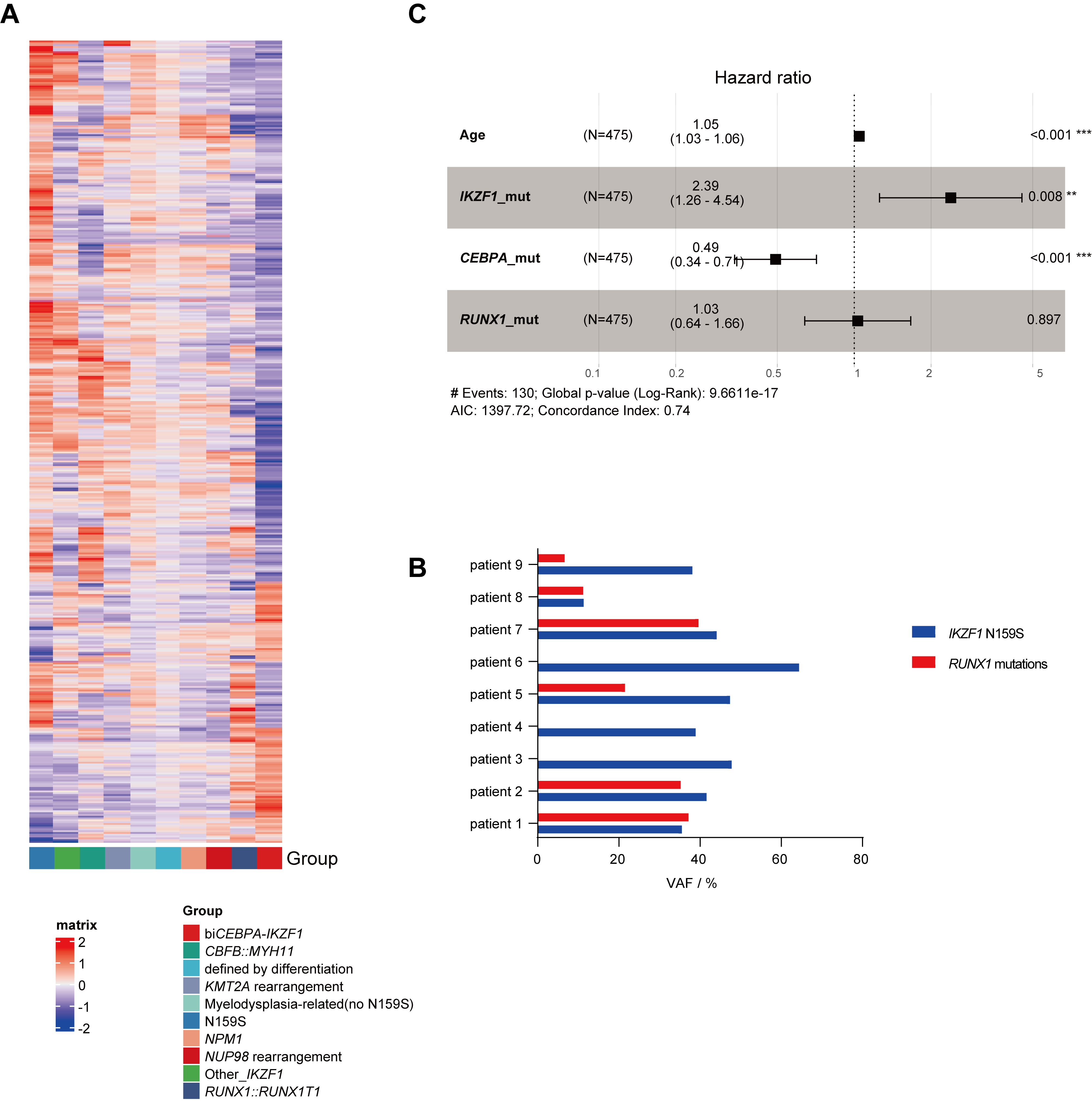

Supplement: Supplementary file 5 — Supporting Information [file CTM2-13-e1309-s005.tif]

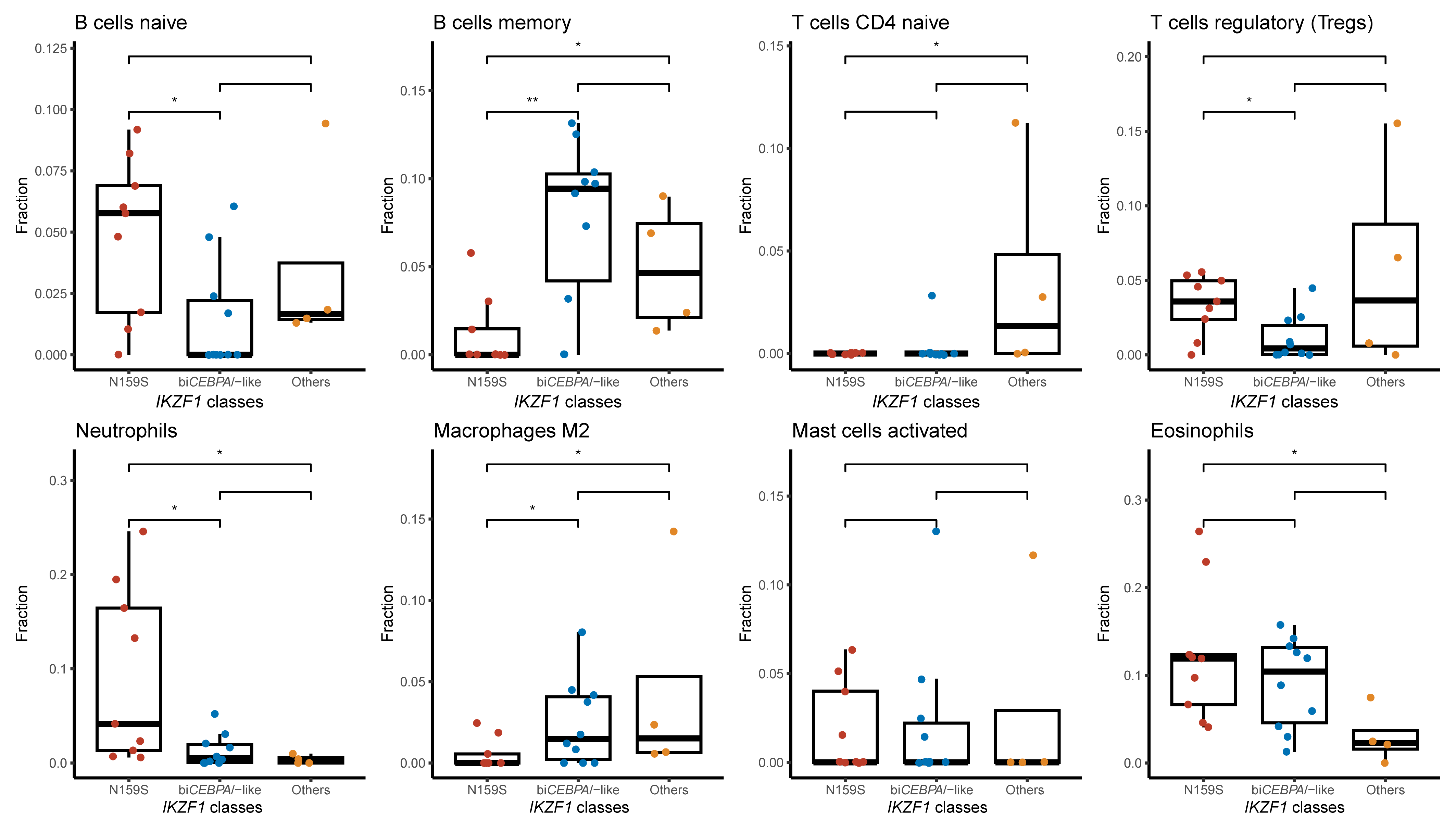

Supplement: Supplementary file 6 — Supporting Information [file CTM2-13-e1309-s006.tif]

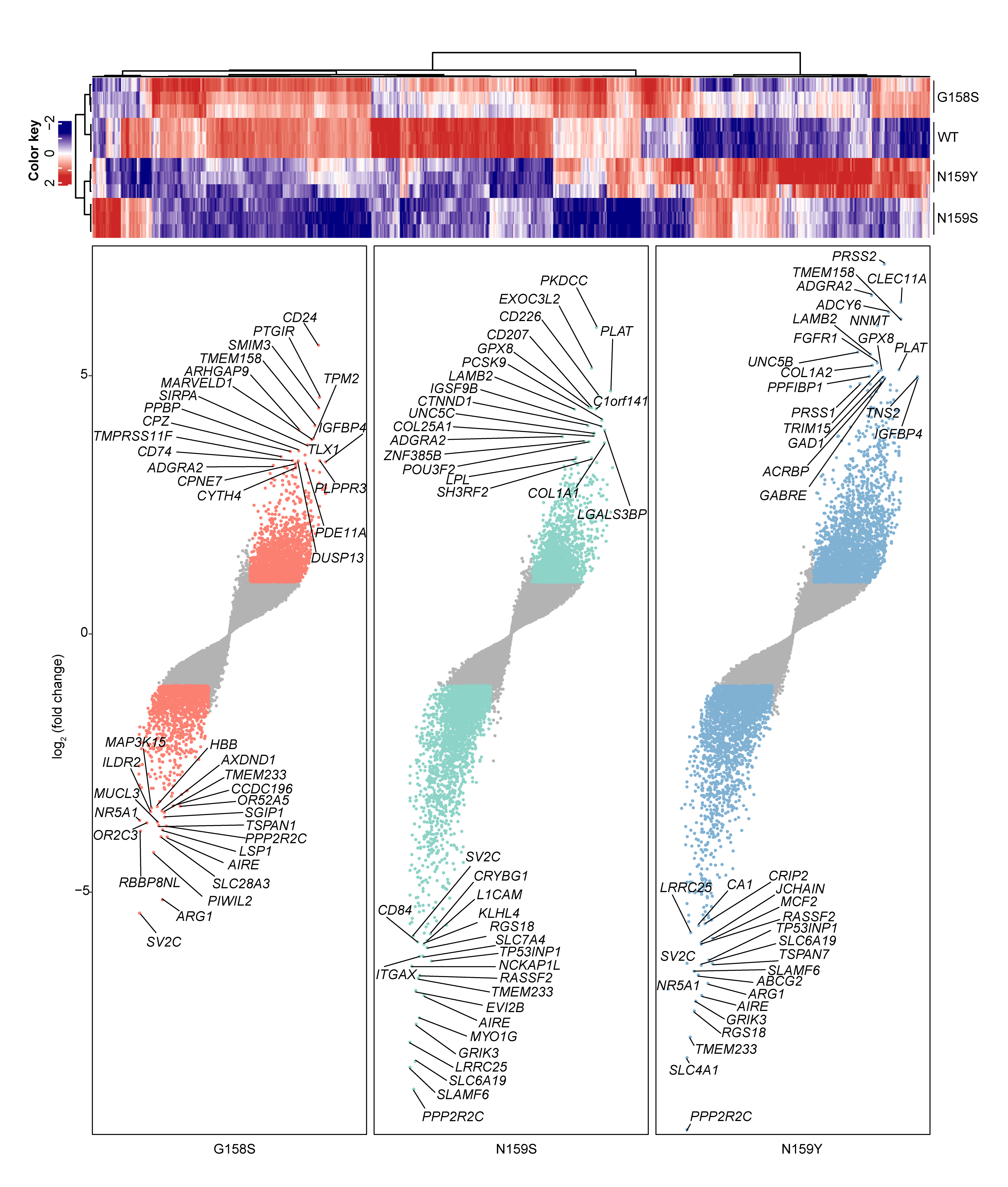

Supplement: Supplementary file 7 — Supporting Information [file CTM2-13-e1309-s002.tif]

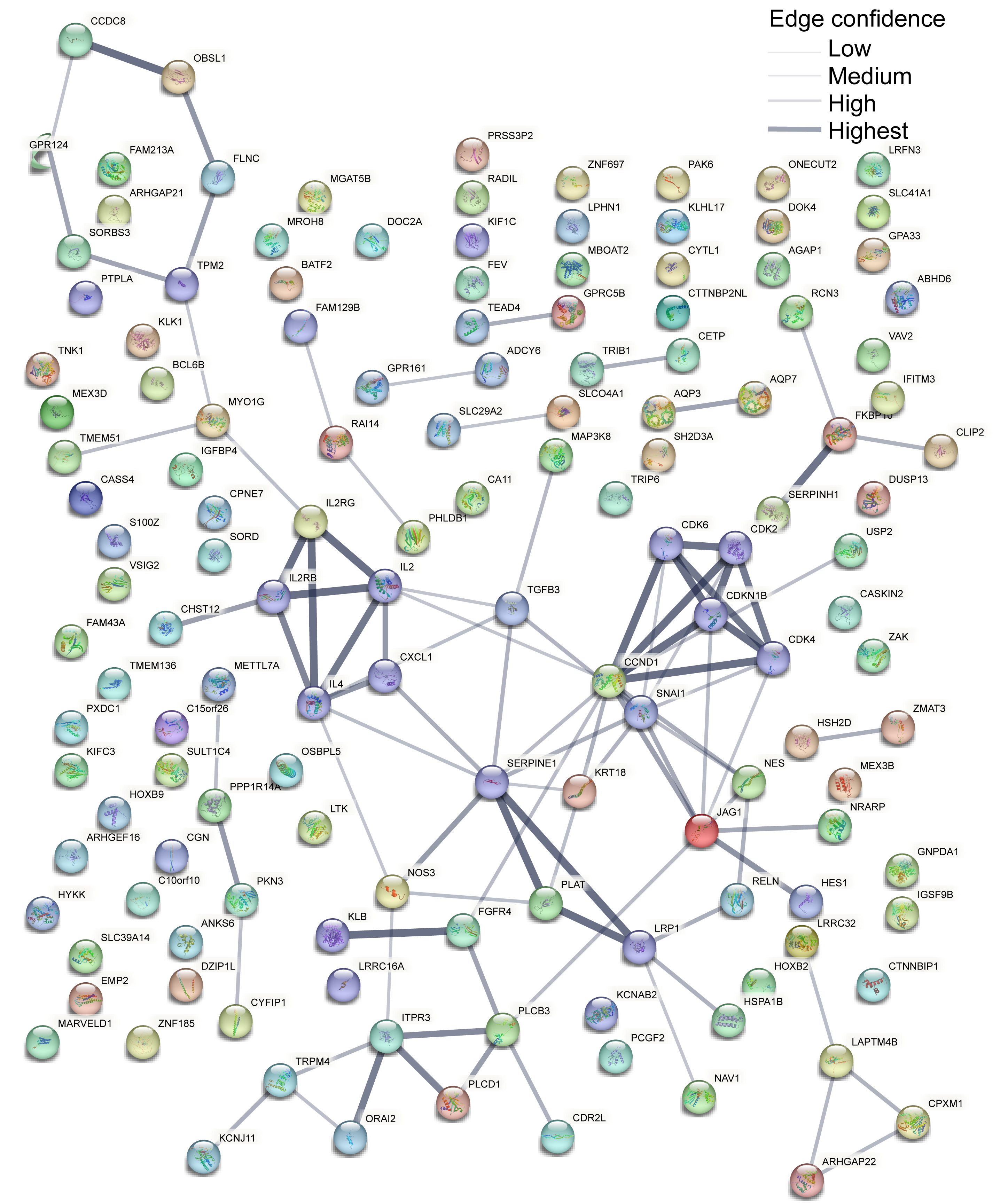

Supplement: Supplementary file 8 — Supporting Information [file CTM2-13-e1309-s004.tif]
