# Supplementary material for: Identification of IKZF1 genetic mutations as new molecular subtypes in acute myeloid leukaemia
Source: Clin Transl Med. 2023 Jun 21;13(6):e1309. doi: 10.1002/ctm2.1309 (PMC10285267; doi:10.1002/ctm2.1309)
Supplement: Supplementary file 9 — Supporting Information [file CTM2-13-e1309-s001.docx]

Supplementary Information Text

SI Materials and Methods

Cells

Human AML cell lines K562 (serial: TCHu191, RRID: CVCL_5J01), U937 (RRID:CVCL_Y171), 293T cell (serial: GNHu17, RRID: CVCL_XY89) and NCI-H1299(serial: SCSP-589, RRID: CVCL_0060) were obtained from National Collection of Authenticated Cell Cultures. K562 and U937 were cultured in RPMI 1640 (BasalMedia, L210KJ) with 10% FBS (Hyclone, SH30406.05) and 1% Penicillin Streptomycin (Gibco, 15140-122). 293T and NCI-H1299 were cultured in DMEM (BasalMedia, L110KJ) with 10% FBS (Hyclone, SH30406.05) and 1% Penicillin Streptomycin (Gibco, 15140-122). All cells were maintained at 37 ℃/5% CO2.

## Lentivirus packaging and transduction

Expression plasmids of various IKZF1 were constructed by inserting the corresponding cDNA sequences into the MIGR1 vector. 11·25 μg of the IKZF1 wild-type or mutation construct, 11·25 μg of the Gag-pol package plasmid and 7·5 μg of the VSV-G envelop plasmid were co-transduced into HEK-293T cells of 75% density in a 10cm dish using lipofectamine 2000 (Invitrogen™Lipofectamine™ 2000 Transfection Reagent, 11668027), and lentiviral particles were collected 48 and 72 hours after transfection, centrifuged and filtered. After that, lentivirus was added to target cells K562 and U937 with 8μg/mL polybrene (Sigma-Aldrich, TR-1003), and transduced cells were verified and selected by flow cytology and western blot.

shRNA-expression vector used in our experiment was pLKO.1. 15 μg of the pLKO.1-shRNA construct, 9 μg of the psPAX2 package plasmid and 6 μg of the pMD2G envelop plasmid were co-transduced into HEK-293T cells of 75% density in a 10cm dish by lipofectamine 2000 (Invitrogen™Lipofectamine™ 2000 Transfection Reagent, 11668027), and lentiviral particles were harvested 48 and 72 hours after transfection, Also, lentivirus was used to infect our target cells IKZF1 N159S expressing K562 with 8μg/mL polybrene (Sigma-Aldrich, TR-1003), and gene-knockdown cells were verified mainly by qPCR and western blot.

The target sequences of shRNA were listed below.

| **target sequences of shRNA** | |
| --- | --- |
| shCPNE7-1 | GATCG-CCATGACTTTGCCATCAATTTCTCGAGAAATTGATGGCAAAGTCATGG-TTTTTT |
| shCPNE7-2 | GATCG-CTTCACGGTGGACTACTACTTCTCGAGAAGTAGTAGTCCACCGTGAAG-TTTTTT |
| Shc-MYC-1 | GATCG-CCTGAGACAGATCAGCAACAATTCAAGAGATTGTTGCTGATCTGTCTCAGG-TTTTTT |
| Shc-MYC-2 | GATCG-CAGTTGAAACACAAACTTGAATTCAAGAGATTCAAGTTTGTGTTTCAACTG-TTTTTT |

## Quantitative RT-PCR

Total RNA of IKZF1-expressing K562 cells and gene-knockdown IKZF1 N159S expressing K562 cells were extracted using the RNeasy Mini Kit (Qiagen,74104) following the instruction manual. cDNA was generated using the HiScript® III 1st Strand cDNA Synthesis Kit (Vazyme, R312-01) from 1 μg total RNA. 1:10 diluted cDNA was used for qPCR analysis, qPCR was performed under the instruction of ChamQ SYBR qPCR Master Mix (Vazyme, Q331-012) with ABI ViiA 7 Real-Time PCR System. The relative expression level of each gene was calculated as 2-△△Ct normalized to the expression of GAPDH.

Primer sequences utilized for qPCR were listed below.

| **Primer sequences utilized for qPCR** | |  |
| --- | --- | --- |
| IKZF1 | F: GCAAAGCTCCAAGAGTGACAGA | R: AGGCACGCCCA TTCTCTTC |
| c-MYC | F: CGTCCTCGGATTCTCTGCTC | R: GCTGGTGCATTTTCGGTTGT |
| CPNE7 | F: GACCTCTTCAGCAAGTCCGAC | R: CACACCGGGTTCAGGTTGT |
| CPNE8 | F: CAATGGTCGAATTGGATGGAGA | R: GCCATGCTCAGTATGTGGTTTC |
| GAPDH | F: CTGGTAAAGTGGATATTGTTGCCAT | R: TGGAATCATATTGGAACATGTAAACC |

## Western Blot

Total cell lysates were prepared under the instruction of Sigma Sample Buffer, Laemmli 2* (SIGMA-ALDRICH, S3401). Protein extracts were quantified using Enhanced BCA Protein Assay Kit (Beyotime, P0009) and analyzed by SDS–polyacrylamide gel electrophoresis and western blotting.

antibody utilized for Western Blot were listed as following:

| Monoclonal ANTI-FLAG M2 antibody | SIGMA-ALDRICH, F1804 | RRID: AB_262044 |
| --- | --- | --- |
| Monoclonal ANTI-Ikaros (D6N9Y) Rabbit antibody | CELL SIGNALING TECHNOLOGY, 14859 | RRID: AB_2744523 |
| Monoclonal Anti-c-MYC antibody | SIGMA-ALDRICH, M4439 | RRID: AB_439694 |
| CPNE7 polyclonal antibody | GENXSPAN, GXP117695 |  |
| CPNE8 polyclonal antibody | BIOWORLD, BS65126 |  |
| Anti-rabbit IgG, HRP-linked Antibody | CELL SIGNALING TECHNOLOGY, 7074 | RRID: AB_2099233 |
| Anti-mouse IgG, HRP-linked Antibody | CELL SIGNALING TECHNOLOGY, 7076 | RRID: AB_330924 |

## Flow cytometry of cell apoptosis and cell cycle analysis

Flow cytometry analysis was used to detect the lentiviral Infection efficiency, cell apoptosis and cell cycle changes. cell apoptosis was detected under the instruction manual of eBioscience™ Annexin V Apoptosis Detection Kits (ThermoFisher, 88-8007-74). Annexin-APC and PI were used for apoptosis analysis, cells were washed with binding buffer and then incubated with indicated antibodies for 20 minutes, flow cytometry was performed on LRSII (BD Biosciences, San Jose, CA), and the results were analyzed using FlowJo software. Cell cycle detection was performed following the instruction of Liankebio Cell Cycle Staining Buffer (MULTI SCIENCES, CCS01). Cells were washed by DPBS (BasalMedia, B320KJ) and then incubated with PI staining buffer for 40 minutes, flow cytometry was performed on LRSII (BD Biosciences, San Jose, CA), Also, the results were analyzed using FlowJo software.

## CCK8 proliferation assay

The proliferation of *IKZF1* expressing K562 cells, U397 cells and gene-knockdown *IKZF1* N159S expressing K562 cells were measured by Cell Counting Kit-8 (TOPSCIENCE, C0005) assay according to the manufacturer instructions. Cells were seeded into 96-well plate at the concentration of 2000 cells/well and maintained for 24, 48, 72, 96 and 120 h, CCK-8 was added to each well at a specific timepoint, after that cells were incubated at 37 °C for 2h before measuring fluorescence. The optical densities (ODs) were measured at a wavelength of 450 nm with a microplate reader (Thermo Fisher Scientific). The relative proliferation level was normalized to the proliferation rate of native cells or cells expressing empty vector. Each group of the experiments was conducted in triplicate.

## Luciferase assay

Human CPNE7 promoter and enhancer region (-2000~500) were amplified and cloned into the pGL4.14 vector, human *c-MYC* promoter and enhancer region were amplified and cloned into the pGL3 vector. luciferase constructs, pRL-SV40 renilla plasmid and *IKZF1* WT or *IKZF1* N159S expressing plasmids (as described before) were transduced into NCI-H1299 cells using FuGENE® 6 Transfection Reagent (Promega, E2691). Both firefly luciferase activity and renilla luciferase activity were detected 48 hours after transfection using the Dual-Luciferase Reporter Assay System (Promega, E1910) following the manufacturer instructions in the GloMax 20/20 Luminometer (Promega). Renilla luciferase activity was used as normalization to control the transfection efficiency.

Primer sequences utilized for the construction of Luc-plasmids were listed below.

| **Primer sequences utilized for the construction of Luc-plasmids** | |  |
| --- | --- | --- |
| CPNE7 | F: CCTGAGCTCGCTAGCCTCGAGCGTGTATGTGTGTGGATCTCATTTC | R: CAGTACCGGATTGCCAAGCTTTGAGGGGCCGCTGCCCGCGAGTC |
| c-MYC | F: CCGCTCGAGTGTGCCTGGAAAAGTCATCA | R: CGTAAGCTTGGAGTGCAGTGGTGCAACTA |

## DNA/RNA sequencing library and platform

Patient’s bone marrow (BM) mononuclear cells were employed for extracting genomic DNA and total RNA using AllPrep DNA/RNA Mini Kit (Qiagen) or TRIzol reagent (Invitrogen) under manufacturer’s instructions. DNA/RNA quality and quantity were assessed by Agilent 2100 Bioanalyzer system (Agilent Technologies) and Qubit (Life Technologies) respectively before library construction. Sequencing library construction of RNA(RNA-Seq) and whole exome sequencing (WES) were implemented using the KAPA RNA HyperPrep kit (Roche) and SeqCap EZ Human Exome v3.0 kit (Roche) respectively. RNA library and WES library then sequenced on the NovaSeq 6000 platform (Illumina) following manufacturer’s instruction.

Cell line total RNA was extracted under the manufacturer’s instruction of the RNeasy Mini Kit (Qiagen,74104). RNA quantification and integrity were assessed using the NanoDrop 2000 spectrophotometer (Thermo Scientific, USA) and the Agilent 2100 Bioanalyzer (Agilent Technologies, Santa Clara, CA, USA) respectively. Sequencing libraries were prepared using TruSeq Stranded mRNA LT Sample Prep Kit (Illumina, San Diego, CA, USA) according to the manufacturer’s protocol. The transcriptome sequencing and analysis were conducted by OE Biotech Co., Ltd. (Shanghai, China).

Hybrid capture-based targeted exome sequencing (TES) was conducted on the coding region of 100 genes involved in acute leukemia and the library enrichment of TES was implemented following the instruction of NadPrep EZ DNA Library Preparation Kit (Nanodigmbio), and sequencing was carried out on a NextSeq 550 platform (Illumina).

## High throughput CUT&Tag sequencing

Shanghai Jiayin Biotechnology Has assisted us in conducting the CUT&Tag experiments. Specific assay was performed following Hatice et al modifications ^1^. One hundred thousand cells of each sample were used to conducte the experiment, after washing with wash buffer(20 mM HEPES pH 7·5; 150 mM NaCl; 0·5 mM Spermidine; 1× Protease inhibitor cocktail), 10μL Concanavalin A coated magnetic beads (Bangs Laboratories) were incubated with each sample for 10min at RT, cells bound with beads were collected and resuspended by dig wash buffer (20mM HEPES pH 7·5; 150mM NaCl; 0·5mM Spermidine; 1× Protease inhibitor cocktail; 0·05% Digitonin; 2mM EDTA). Primary antibody and IgG control antibody (Monoclonal ANTI-FLAG M2 antibody, SIGMA-ALDRICH, F1804; normal mouse IgG, Millipore, 12-371) were added and the samples were incubated on a rotating platform overnight at 4°C.Day2 secondary antibody (Rabbit Anti-Mouse IgG H&L, abcam, ab611709) was added into cells and co-incubated at RT for 1 hour. After being washed by dig wash buffer, cells were incubated with pA-Tn5 adapter complex prepared in dig-med buffer (0·01% Digitonin; 20mM HEPES pH 7·5; 300mM NaCl; 0·5mM Spermidine; 1× Protease inhibitor cocktail) for 60 minutes in RT. Finally, cells were collected and resuspended in tagmentation buffer (10mM MgCl2 in Dig-med Buffer) and DNA was purified by phenol-chloroform-isoamyl alcohol extraction and ethanol precipitation.

DNA libraries were amplified using a universal i5 and a barcoded i7 primer through PCR cycling conditions: 72 °C for 5 min (gap filling); 98 °C for 30 s; 14 cycles of 98 °C for 10 s and 63 °C for 30 s; extension at 72 °C for 1 min and hold at 8 °C by NEBNext HiFi 2× PCR Master mix. libraries clean-ups were performed XP beads. (Beckman Counter)

The size distribution of libraries was determined by Agilent 4200 TapeStation analysis, and Sequencing was performed in the Illumina Novaseq 6000 using 150bp paired end following the manufacturer’s instructions.

## Variant calling of targeted sequencing data

The BWA (v 0.7.17-r1188) program aligned paired reads to the hg19 reference genome. GATK was used to pre-process the aligned reads for downstream variant calling ^2^. The SNVs and INDELs of TES were called using combined GATK4 Mutect2, VarDict (v1.5.8) ^3^ and MuTect (v1.1.7) programs. Then, reported variants were annotated using snpEff (v4.2) ^4^ and ANNOVAR (v2019Dec03). Detailed filtration steps were described in our previous work ^2^.

## Gene expression quantification

The alignment strategy-based approach for gene expression quantification begins with aligning raw RNA-Seq reads to the human hg38 reference genome using the two-pass mode of STAR (v2.7.9a) ^5^ and the gene model of GENECODEv38. Afterward, transcript or gene expression counts were extracted from the aligned BAM files using Featurecounts (v2.0.1) ^6^. Data normalization and variance-stabilizing transformation (VST) were performed using DESeq2 (v1.28.0) ^7^. ComplexHeatmap (v2.12.0) ^2^, iMeta) and ward.D algorithm were used to conducte hierarchical clustering. The clustering distance measure was defined by the ‘as.dist((1-cor(t(x)))/2)’ function. The limma (v3.52.2) ^2^ program was used to generate the list of differentially expressed genes. String website (https://cn.string-db.org) was used to construct the protein-protein interaction network. All interaction sources were retained in the network analysis. The highest confidence (0·9) cutoff was used to construct the network of DEGs of *IKZF1* N159S-positive patients, while the low confidence (0·15) cutoff for displaying more connected relationships using the intersection of *IKZF1* target genes and DEGs of patients and cell line. The CIBERSORTx online website was used to calculate the fractions of 22 human hematopoietic cell phenotypes ^8^. The single sample gene set enrichment analysis (ssGSEA) algorithm ^9^ and reported gene sets ^10^ were used to quantify the enrichment of three different developmental stages of myeloid cells including human hematopoietic stem and progenitor cell (HSPC), granulocytes-macrophage (GMP) and monocyte.

## Gene fusion and tandem duplication analysis

RNA-Seq data was used to identify potential fusion genes, while *FLT3*-internal tandem duplication (ITD) and *KMT2A*-partial tandem duplication (PTD) were detected based on targeted sequencing and Sanger sequencing. Chimeric transcripts were reported by Fusioncatcher (v1.20) ^11^ and Arriba (v2.0.0) ^12^ programs following strict filtration steps ^2^.

## Genomic binding analysis of IKZF1 N159S and WT IKZF1

The nextflow-based pipeline nf-core/cutandrun (version 81ede3365d) and its Singularity container were used to conduct the quality control steps and reads alignments of CUT&RUN sequencing data. It wrapped Trim Galore to clean the CUT&RUN raw sequencing reads and aligned the reads to hg38 genome using Bowtie2 program. Picard was used to marke duplicated reads in aligned BAM files. The bedGraph and bigWig files were generated based on bedtools and bedGraphToBigWig programs, respectively. The SICER program was used to infer the differential binding peaks between IKZF1 N159S and WT IKZF1. The homer (v4.11.1) was used to annotate the peaks list. The deepTools (v3.5.1) sub-programs computeMatrix and plotHeatmap were used to draw the differentially binding profiles (FDR < 0·01) in heatmap. The IGV (v2.10) program was used to visualize the genomic binding tracks.

SI Figures


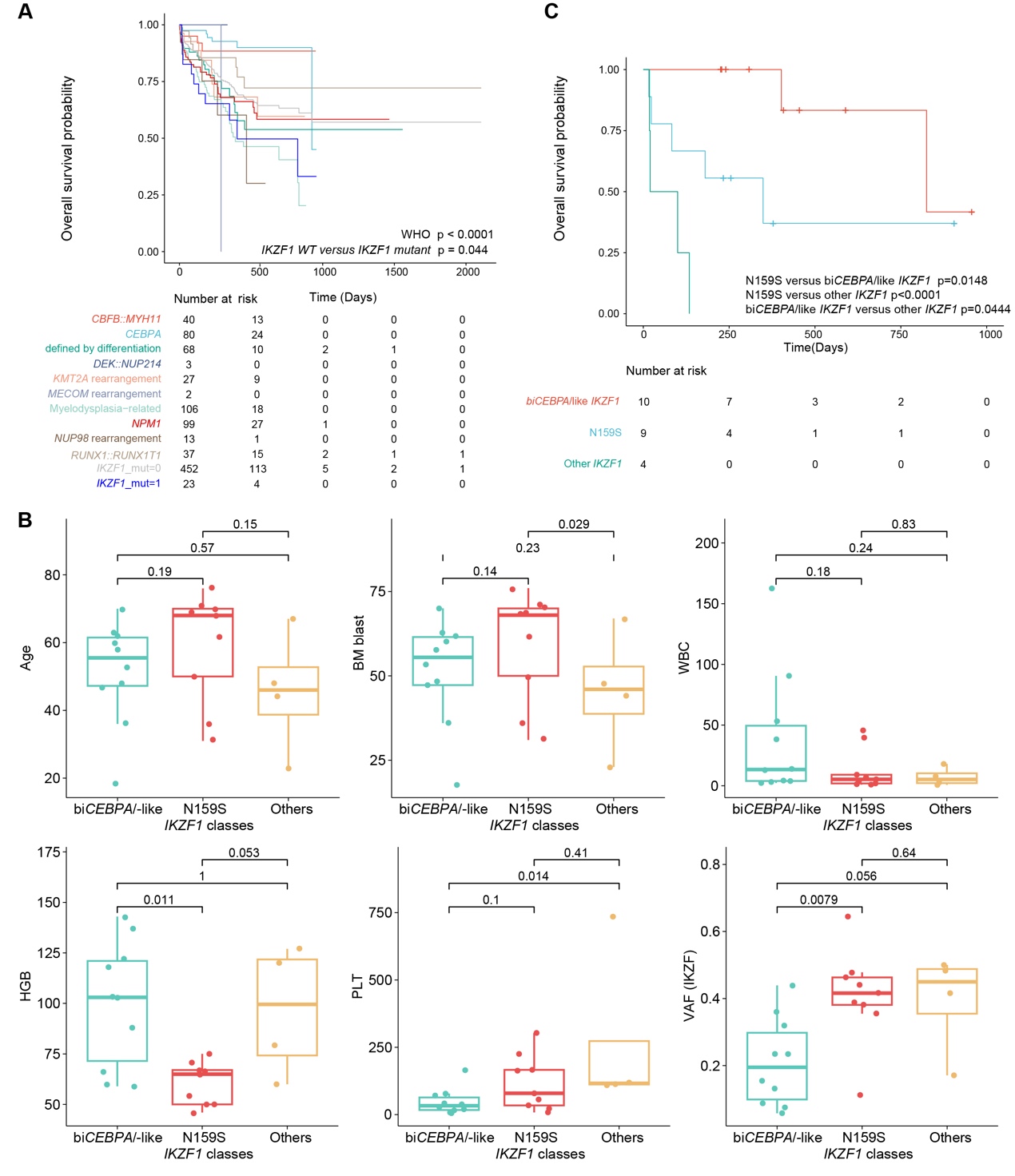


**Figure S1. Clinical feature of IKZF1-positive patients: (A)** Overall survival of our patients with AML according to the 5th edition of the World Health Organization (WHO) Classification and *IKZF1* mutation status. **(B)** Violin diagram depicted the clinical feature difference among patients with *IKZF1* N159S, bi*CEBPA*/-like IKZF1 and others. **(C)** Overall survival of patients with AML according to *IKZF1* mutation classes, Kaplan–Meier curves depicting the survival difference between biCEBPA/-like patients (red line), N159S-mutated patients (blue line), and other patients (green line).

**Figure S2. HSPC enrichment score of *IKZF1*-positive patients:** Box diagram demonstrated enrichment score of HSPC, GMP and Monocyte signatures among *IKZF1*-positive patients.


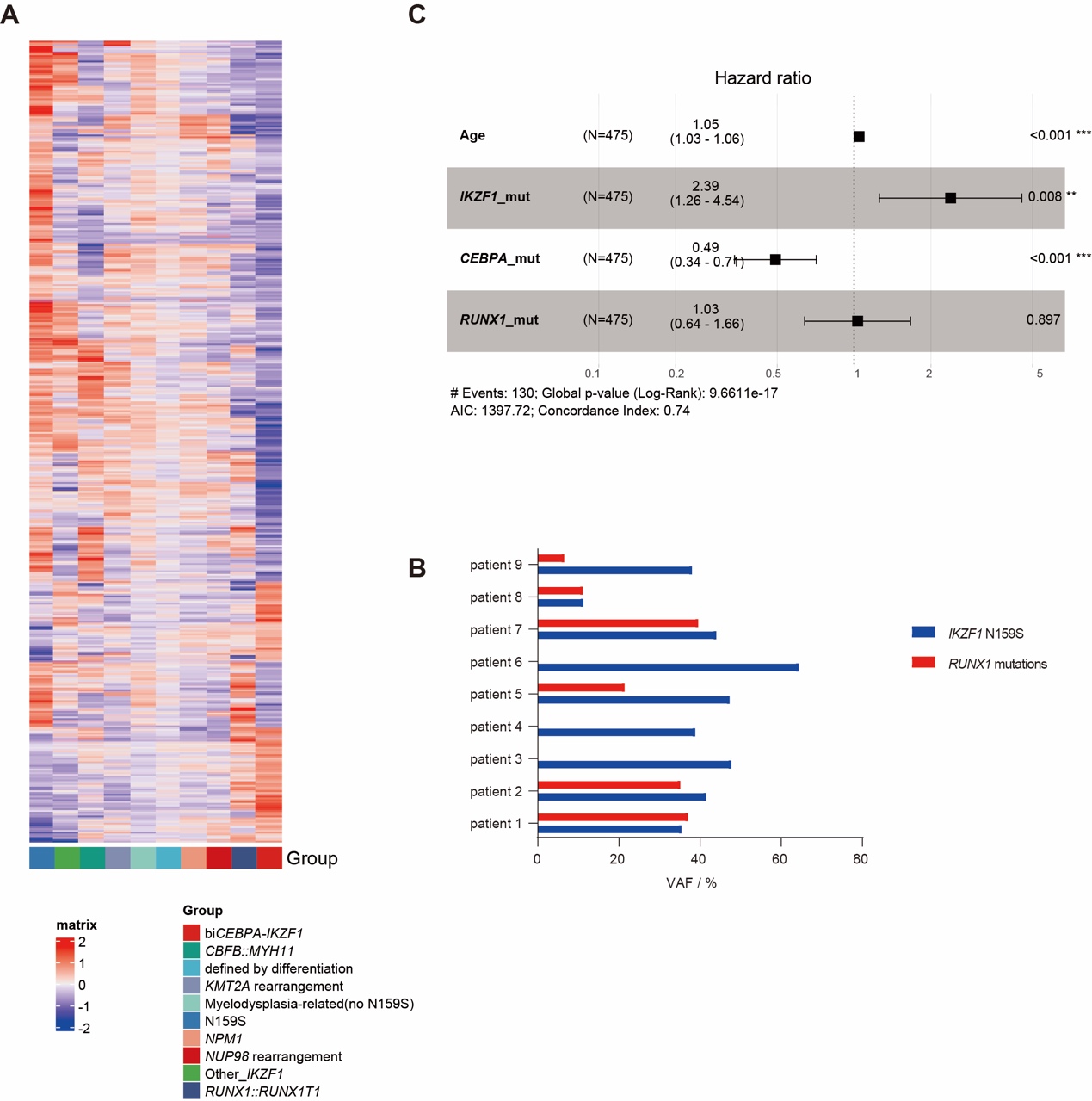


**Figure S3. *IKZF1* N159S defines a rare molecular subtype with unique** **gene expression profiles in AML: (A)** Gene expression clustering comparison between *IKZF1*-mutated AML and WHO classification-based AML using the same 2308 variance genes with p-value less than 0·05 confirmed the genomic-based subgroup of *IKZF1*-N159S AML. **(B)** Variant allele frequency (VAF) comparison between *IKZF1*-N159S mutation and *RUNX1* mutation in *IKZF1* N159S-positive cases. **(C)** Cox regression analysis identified that age, *IKZF1* status and *CEBPA* status could be independent factors for OS in our AML cohort, while RUNX1 status cannot be a prognostic predictor.

**Figure S4. immune cells fraction of *IKZF1*-positive patients:** Box diagram demonstrated immune cells fraction including native B cells, T cells regulatory (Tregs) and neutrophils among *IKZF1*-positive patients.

**Figure S5. cell line unsupervised. DEGs:** Unsupervised clustering of differentially up- and down-regulated expressed genes (DEGs) between *IKZF1* G158S, N159S and N159Y mutation knock-in human cell lines (versus WT).


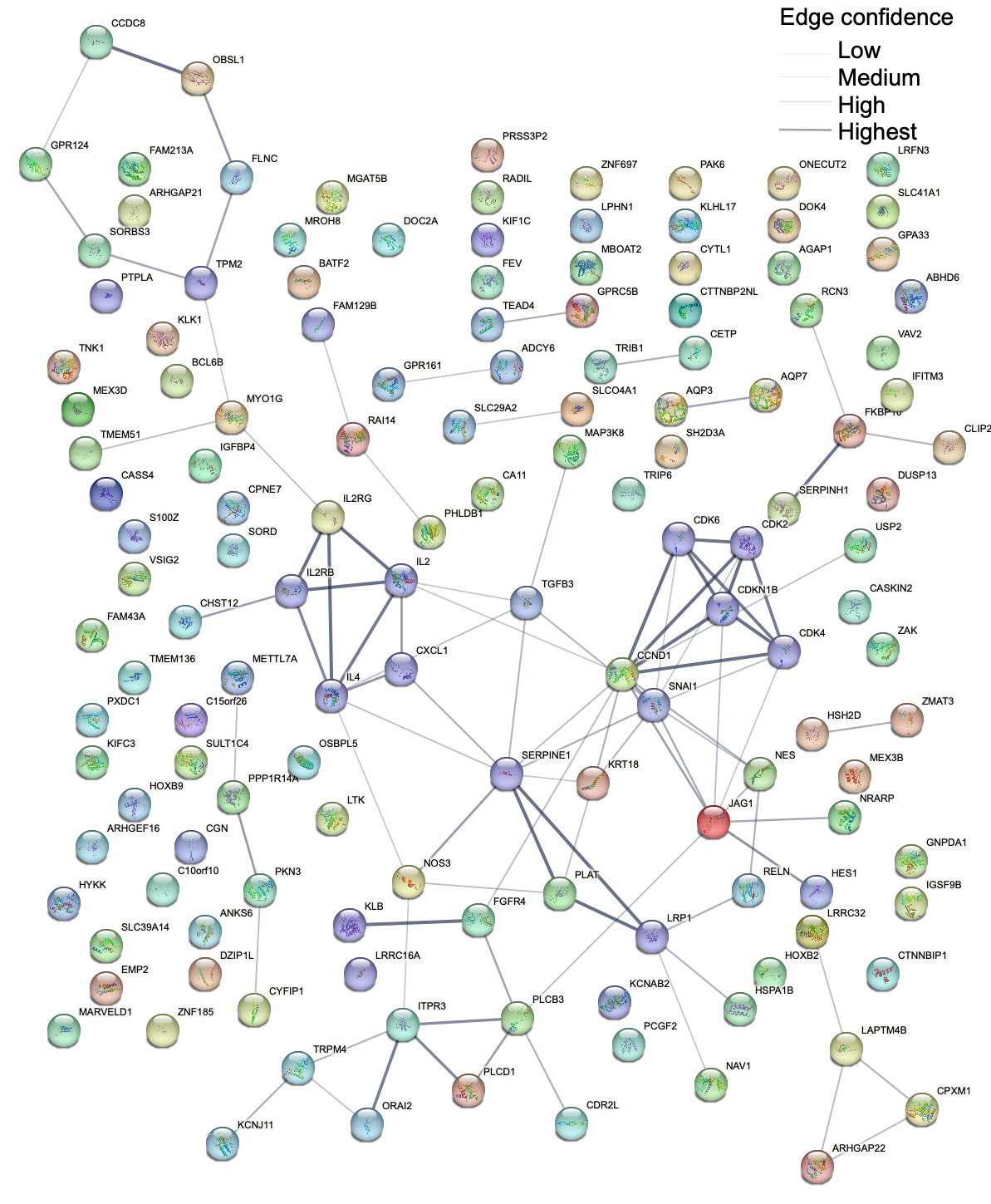


**Figure S6.** **Protein-protein interaction (PPI) network based on intersection gene sets using *IKZF1* N159S target genes:** A protein interaction network was constructed to integrate and visualize the up- and down-regulated genes of both *IKZF1* N159S-positive AML patients and *IKZF1* N159S knock in human cell line.

SI Tables

**Table S1 summary of AML patients with IKZF1 mutation**

**Table S2 bulk RNA-Seq and CUT&TAG sequencing of human cell lines**

**Reference**

Uncategorized References

1. Kaya-Okur HS, Wu SJ, Codomo CA, Pledger ES, Bryson TD, Henikoff JG, et al. CUT&Tag for efficient epigenomic profiling of small samples and single cells. Nat Commun. 2019;10(1):1930.

2. Cheng WY, Li JF, Zhu YM, Lin XJ, Wen LJ, Zhang F, et al. Transcriptome-based molecular subtypes and differentiation hierarchies improve the classification framework of acute myeloid leukemia. Proc Natl Acad Sci U S A. 2022;119(49):e2211429119.

3. Lai ZW, Markovets A, Ahdesmaki M, Chapman B, Hofmann O, McEwen R, et al. VarDict: a novel and versatile variant caller for next-generation sequencing in cancer research. Nucleic Acids Research. 2016;44(11).

4. Cingolani P, Platts A, Wang le L, Coon M, Nguyen T, Wang L, et al. A program for annotating and predicting the effects of single nucleotide polymorphisms, SnpEff: SNPs in the genome of Drosophila melanogaster strain w1118; iso-2; iso-3. Fly (Austin). 2012;6(2):80-92.

5. Dobin A, Davis CA, Schlesinger F, Drenkow J, Zaleski C, Jha S, et al. STAR: ultrafast universal RNA-seq aligner. Bioinformatics. 2013;29(1):15-21.

6. Liao Y, Smyth GK, Shi W. featureCounts: an efficient general purpose program for assigning sequence reads to genomic features. Bioinformatics. 2014;30(7):923-30.

7. Love MI, Huber W, Anders S. Moderated estimation of fold change and dispersion for RNA-seq data with DESeq2. Genome Biology. 2014;15(12).

8. Newman AM, Steen CB, Liu CL, Gentles AJ, Chaudhuri AA, Scherer F, et al. Determining cell type abundance and expression from bulk tissues with digital cytometry. Nat Biotechnol. 2019;37(7):773-82.

9. Hanzelmann S, Castelo R, Guinney J. GSVA: gene set variation analysis for microarray and RNA-seq data. BMC Bioinformatics. 2013;14:7.

10. van Galen P, Hovestadt V, Wadsworth Ii MH, Hughes TK, Griffin GK, Battaglia S, et al. Single-Cell RNA-Seq Reveals AML Hierarchies Relevant to Disease Progression and Immunity. Cell. 2019;176(6):1265-81 e24.

11. Edgren H, Murumagi A, Kangaspeska S, Nicorici D, Hongisto V, Kleivi K, et al. Identification of fusion genes in breast cancer by paired-end RNA-sequencing. Genome Biology. 2011;12(1).

12. Uhrig S, Ellermann J, Walther T, Burkhardt P, Frohlich M, Hutter B, et al. Accurate and efficient detection of gene fusions from RNA sequencing data. Genome Res. 2021;31(3):448-60.
